# Highly parallel lab evolution reveals that epistasis can curb the evolution of antibiotic resistance

**DOI:** 10.1038/s41467-020-16932-z

**Published:** 2020-06-19

**Authors:** Marta Lukačišinová, Booshini Fernando, Tobias Bollenbach

**Affiliations:** 1 University of Cologne, Institute for Biological Physics, Zülpicher Straße 77, 50937 Cologne, Germany; 2 IST Austria, Am Campus 1, 3400 Klosterneuburg, Austria; 3 Present Address: Department of Biology, Technion – Israel Institute of Technology, Haifa, 32000 Israel

**Keywords:** Experimental evolution, Antibiotics, Antimicrobial resistance, Evolvability

## Abstract

Genetic perturbations that affect bacterial resistance to antibiotics have been characterized genome-wide, but how do such perturbations interact with subsequent evolutionary adaptation to the drug? Here, we show that strong epistasis between resistance mutations and systematically identified genes can be exploited to control spontaneous resistance evolution. We evolved hundreds of *Escherichia coli* K-12 mutant populations in parallel, using a robotic platform that tightly controls population size and selection pressure. We find a global diminishing-returns epistasis pattern: strains that are initially more sensitive generally undergo larger resistance gains. However, some gene deletion strains deviate from this general trend and curtail the evolvability of resistance, including deletions of genes for membrane transport, LPS biosynthesis, and chaperones. Deletions of efflux pump genes force evolution on inferior mutational paths, not explored in the wild type, and some of these essentially block resistance evolution. This effect is due to strong negative epistasis with resistance mutations. The identified genes and cellular functions provide potential targets for development of adjuvants that may block spontaneous resistance evolution when combined with antibiotics.

## Abstract (web summary)

The antibiotic resistance crisis calls for new ways of restricting the ability of bacteria to evolve resistance. Here, Lukačišinová et al. perform highly controlled evolution experiments in *E. coli* strains to identify genetic perturbations that strongly limit the evolution of antibiotic resistance through epistasis.

## Introduction

Bacterial resistance to antibiotics has become a major public health concern and a vibrant field of research^1–3^. Strategies for countering the spread of resistance include the discovery of new antibiotics and drug combinations^4–6^. Given the increasing difficulty of identifying new drugs, recent work has focused on novel treatment schemes that minimize selection for resistance using drug cycling or combinations that exploit physiological or evolutionary interactions between drugs^7–12^. More sustainable drug treatments, however, require novel strategies that anticipate the evolutionary potential of pathogens and funnel them toward less evolvable genotypes or evolutionary dead ends. To this end, it is promising to identify genetic factors and cellular mechanisms that do not immediately increase a pathogen’s resistance but rather determine its ability to evolve^13–16^. The possibility of interfering with the ability of different genotypes to spontaneously evolve drug resistance (here: “resistance evolvability”) by lowering the mutation rate or the rate of horizontal gene transfer has been investigated in depth^13,17–21^.

Clinical antibiotic resistance is often due to horizontally transferred resistance genes; a case in point is tetracycline resistance, which is mediated primarily by drug efflux proteins encoded by the plasmid-borne resistance genes *tetA* and *tetB*^22^. When such resistance genes are unavailable, antibiotic resistance can evolve by spontaneous mutations. For example, mutations can enhance expression of chromosomally encoded multidrug efflux pumps, leading to moderate-level resistance^23^. Alternatively, high-level resistance for specific antibiotics such as quinolones can be gained by mutations in the drug target, preventing antibiotic binding^24^. In this case, increased mutation rates can accelerate resistance evolution^25^. Consequently, many studies have focused on identifying ways of slowing resistance evolution by preventing an increase of the mutation rate under stress (stress-induced mutagenesis)^13–15,26,27^.

There have been notable successes in preventing stress-induced mutagenesis^13–15,17^. However, the mutation rate cannot be lowered indefinitely due to biophysical limits^28^. As a result, the potential of slowing resistance evolution by approaches focused on mutation rate is fundamentally limited. Efforts to alter evolvability by other means, including the exploitation of genetic interactions, could circumvent this fundamental limit but have received less attention^16,29^. Different epistasis patterns—ways in which genetic differences affect the fitness effects of new mutations—are commonly found in evolution experiments^30,31^. In particular, global patterns of diminishing-returns epistasis, where new mutations produce smaller fitness gains on fit relative to unfit backgrounds, have been observed^32–34^. Genetic interactions can also quantitatively affect antibiotic resistance. Interfering with different cellular functions can sensitize or protect cells^35^ and thus alter the initial state in resistance evolution. But how do such targeted perturbations of cellular functions interact with the subsequent evolutionary adaptation to drugs?

The genetic background determines the effects of resistance mutations, which can enable or block specific mutational paths to drug resistance. For example, the transcriptional regulator AmpR opens a key path to resistance in *Pseudomonas*, since only strains that carry the gene rapidly evolve ceftazidime resistance by overexpressing beta-lactamase^16^. Similar perturbations to transcriptional regulators could alter resistance evolvability more generally, since they can completely change the expression state of the cell, and thus enable selection to act on downstream expressed genes. In fact, perturbing any cellular function that interacts with a resistance mechanism could affect the cell’s ability to evolve. This could be by interfering with its regulation, protein folding, localization, function, or degradation, all of which are potential sources of epistasis. To more generally identify such epistasis patterns and discover mechanisms that determine resistance evolvability, a systematic investigation of resistance evolution starting from a diverse set of defined genotypes is needed.

Resistance evolvability can be measured by exposing bacteria with different genotypes to equivalent selection pressures in evolution experiments and comparing the evolutionary outcomes^36^. Differing population size, selection pressure and the number of generations a population undergoes strongly influence the outcome^37,38^, but these factors often vary due to the inflexibility of the experimental protocol rather than a meaningful difference in evolutionary potential of the starting genotype. A particular challenge in evolution experiments selecting for drug resistance is that the drug concentration needs to be carefully adjusted for each strain. Otherwise, strains that are initially more sensitive can be quickly wiped out and the experiment no longer informs on their ability to evolve. For a sound quantitative comparison, it is essential to control these factors tightly—a characteristic that is achievable due to recent technological advances^39,40^, albeit in relatively low throughput. The outcome of such evolution experiments then depends solely on the evolutionary determinants of the starting genotype, including its mutation rate and the distribution of fitness effects of resistance mutations.

Here, we solved these critical technical issues and report the systematic discovery of targeted genetic perturbations that drastically affect spontaneous antibiotic resistance evolution due to strong epistatic interactions with resistance mutations. We developed a feedback-controlled robotic platform for high-throughput lab evolution, which tightly controls both population size and selection pressure for drug resistance. Quantifying the evolvability of ~100 *E. coli* K-12 gene-deletion strains from the Keio collection^41^ representing major cellular functions revealed a global trend of diminishing-returns epistasis: Genotypes that were initially more sensitive evolve resistance faster and converge to the same limit of resistance as initially resistant ones. Notably, we identified gene deletions that deviate from this global trend due to strong genetic interactions between specific cellular functions and resistance mutations. In some cases, these interactions entirely prevent the evolution of antibiotic resistance within the time scale of our experiments.

## Results

### High-throughput platform measures resistance evolvability

**Fig. 1:**
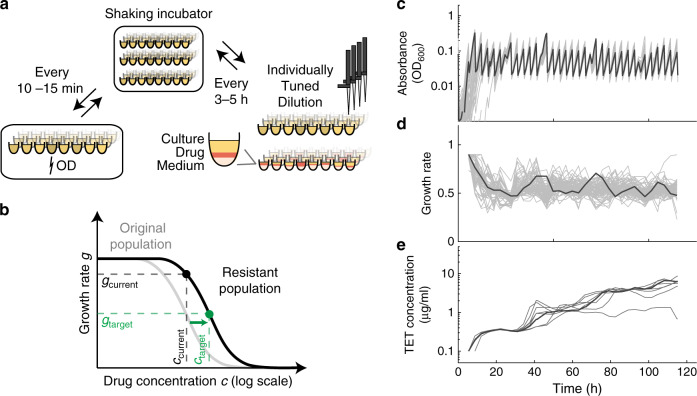
Automated highly controlled lab evolution leads to reproducible fast evolution of antibiotic resistance. **a** Schematic of lab evolution protocol. 96-well plates are shaken in an incubator, optical density (OD) is measured every 10–15 min in a plate reader, and the cultures are diluted and transferred to new plates every 3–5 h. At dilution, the volumes of culture, drug, and medium are individually tuned based on the previous OD measurements (panel **b**), such that the OD after dilution and the growth rate is always close to the predetermined level (panels **c** and **d**, Methods). **b** Schematic of dose–response curve in ancestral (gray line) and a resistant population (black line). At each dilution, the target antibiotic concentration *c*_target_ is calculated assuming that the effect of resistance is equivalent to reducing the concentration by a factor, i.e., a mere horizontal shift of the semi-logarithmic dose–response curve^44^. The growth rate since last dilution and antibiotic concentration in the given well (*g*_current_ and *c*_current_) then define the curve from which the new target concentration *c*_target_ is calculated. **c** OD values over the course of an evolution experiment for all culture-containing wells from a 96-well plate including 25 different gene-deletion mutants. The cultures are continuously in exponential phase as seen by the linear increase of OD on a log scale. The downward changes in OD are due to dilutions to a target OD. **d** Growth rates from fits to the OD traces in (**c**). Values are normalized to the growth rate of the reference strain in no drug. **e** Tetracycline concentration in the wells of eight replicates of the reference strain (a subset of the wells shown in (**c**) and (**d**)). The increase is adjusted to keep the growth rate of each culture close to 50% of drug-free growth rate, despite resistance evolution. An arbitrary single well is highlighted in black on all three plots (c–e). All values are from plate 1 of experiment M1 (Methods, Supplementary Table 2, Source data).

To quantify the dynamics of resistance evolution for many different genotypes and replicates, we developed an automated platform that monitors the growth of hundreds of bacterial cultures while tightly controlling conditions and key evolution parameters (Fig. 1, see Methods). Similar to the “morbidostat” setup^40,42^, the antibiotic concentration of each culture is periodically adjusted to maintain high selection pressure for antibiotic resistance for up to 2 weeks. Every 3–5 h, a dedicated robotic system dilutes and transfers cultures to new 96-well plates (Fig. 1a). In this transfer step, the volumes of medium, drug, and bacterial culture are individually tuned to keep each culture in exponential phase at 50% growth inhibition with defined population size (Fig. 1b–e, Methods). We keep the cultures in exponential phase under vigorous shaking and continuously adjust the antibiotic concentration to ensure strong selection pressure for fast growth in the presence of the antibiotic. If the bacterial population cannot endure the sustained antibiotic stress (e.g., due to slow accumulation of toxic metabolites in the cell), the antibiotic concentration is automatically decreased until the growth rate has recovered to 50%. In this way, we can directly compare resistance evolution between hundreds of bacterial populations of the same size that undergo the same number of generations and experience the same clearly defined selection pressure.

This lab evolution platform yields a precise and reproducible real-time measure of the quantitative resistance increase for each culture. Typically, resistance is measured by determining the minimum concentration needed to stop growth (MIC) or the concentration needed to inhibit growth by 50% (IC_50_). In our automated platform, a feedback loop continually adjusts the antibiotic concentration to maintain 50% growth inhibition. Therefore, the antibiotic concentration in each well is a direct estimate of the IC_50_ (Methods). Indeed, we validated that this “on-the-fly” measurement of the IC_50_ agrees well with a standard IC_50_ measurement in a drug concentration gradient after the evolution experiment (Pearson’s correlation coefficient *r* = 0.8, Supplementary Fig. 1). Thus, our experimental setup can quantitatively measure changes in resistance (IC_50_) in real time; resistance measured in this way can vary continuously and is not confined to pre-defined discrete concentration steps as in classical dose–response curve measurements.

The strong selection pressure leads to rapid evolution: Tetracycline resistance in the *E. coli* K-12 *ΔlacA* strain from the Keio collection^41^ we used as reference strain in our experiments increases by 10–20-fold within 5 days^40^; the parent strain of the Keio collection (BW25113) behaves identically (Fig. 1e, Supplementary Fig. 2). Note that the lab strains we used do not carry or acquire horizontally transferred tetracycline resistance genes in this assay, but evolve spontaneous tetracycline resistance. While this is not the most common cause of tetracycline resistance in the clinic, it enables investigations of resistance evolution with a focus on quantitative and conceptual aspects^12,40,43^. Despite the fundamental stochasticity of evolution, the observed resistance increase over time is usually reproducible for replicates starting from the same genotype (Fig. 1e), in line with previous reports^40,44^. Whole-genome sequencing of evolved *E. coli* strains confirmed that the mutated genes are also largely reproducible (Methods, Supplementary Fig. 3). For example, in the presence of the antibiotic tetracycline, the genes *marR*, *lon*, and *acrR* are often mutated at the end of the experiment (Supplementary Fig. 3a, Supplementary Data 1), in good agreement with previous observations^22,45,46^. Typical evolved populations have three to four fixed mutations after 10 days (Supplementary Fig. 4). Taken together, our automated platform enables phenotypically and genotypically repeatable resistance evolution for hundreds of parallel populations and is suitable for detecting perturbations that can alter resistance evolvability.

### Resistance evolution exhibits diminishing-returns epistasis

**Fig. 2:**
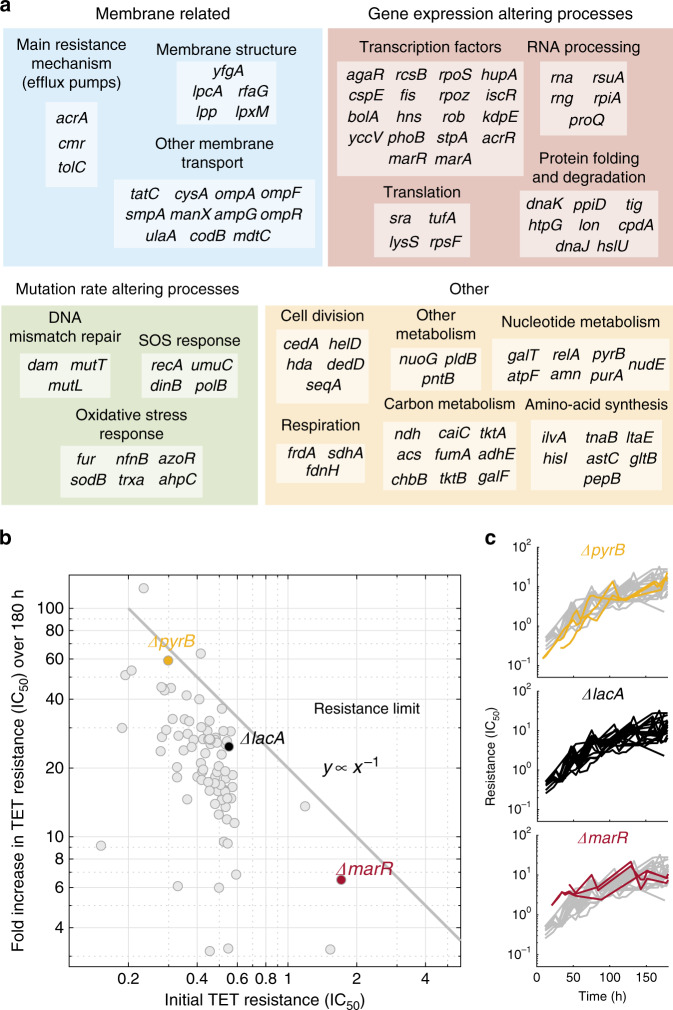
Resistance increases in gene-deletion strains exhibit hallmarks of diminishing-returns epistasis. **a** Gene deletions chosen as ancestors for evolution experiments grouped by possible mechanism of evolvability alteration (large colored boxes) and specific cellular function (small boxes). **b** Mean fold increase in resistance after 180 h of evolution versus initial resistance for each deletion strain. The final and initial resistance measures for each replicate are the mean tetracycline concentrations over appropriate time intervals (12–24 h for initial and 170–180 h for final resistance). The line *y* ∝ *x*^−1^ indicates where the points would lie if all experiments reached the same final resistance irrespective of initial resistance. There is a strong anti-correlation (Spearman’s *ρ* = −0.544, bootstrap 95% confidence interval [−0.735 −0.302], *p* = 2 × 10^−7^) indicating diminishing returns; bias of *ρ* (estimated by bootstrapping) is at most 0.005. **c** Yellow, black, and red lines show resistance change over time for replicates of the three strains highlighted in the same color in (**b**), respectively; resistance change of 22 replicates of the reference strain (*ΔlacA*) are shown in gray for comparison. This figure uses data from experiment M2 in panel **b** and combines data from M2 and M3 in panel **c** (Methods, Supplementary Table 2, Source data).

To address how diverse cellular perturbations affect resistance evolvability, we investigated strains with genetic perturbations in a broad range of cellular functions from the Keio collection, a genome-wide *E. coli* gene-deletion library^41^ (Fig. 2a). Performing such an investigation genome-wide would require over 10,000 evolution experiments (including replicates), which is currently prohibitive, even with the automated evolution platform we developed. Thus, we focused on a sample of 98 gene deletions, which affect diverse cellular functions. We selected more than half of the gene deletions because we hypothesized that they would affect resistance evolvability or they are already known to do so (Methods). Specifically, for 13 out of 98 selected genes, the impaired function could have an effect on evolvability through mutation rate; these functions include DNA-mismatch repair^47,48^, SOS response^13^, and oxidative-stress response^18^. In general, increased mutation rate in these strains is expected to accelerate evolution. Importantly, we further aimed to identify new ways of altering evolvability that are independent of mutation rate changes.

Specifically, we hypothesized that deletions of known resistance genes such as drug efflux pumps^49^ and porins^50,51^, together with several other membrane-related functions (Fig. 2a) can affect resistance evolvability. In particular, as mutations that lead to the overexpression of the AcrAB-TolC multidrug efflux pump are a common way of evolving tetracycline resistance^40,52,53^, it is plausible that disrupting this efflux pump could eliminate the phenotypic impact of these resistance mutations and thus affect resistance evolvability. Perturbations of functions that could systematically interfere with (yet unrecognized) resistance mechanisms were also included, in particular those affecting protein folding^54,55^, membrane composition, and transcription factors (Fig. 2a). Apart from such genes hypothesized to affect resistance evolvability, we included 33 gene deletions that represent diverse cellular pathways expressed in rich medium^35,56^. In general, we selected gene deletions with negligible fitness costs based on control measurements of growth rate in antibiotic-free medium (Supplementary Fig. 5), which is crucial to avoid selecting primarily for suppressor mutations of the gene deletion rather than for drug resistance (Methods); three gene deletions that exhibited greater fitness costs (*rpsF*, *tktA*, *tufA*) were excluded from further analysis. This broad selection of genetically different strains enables the discovery of general trends in resistance evolution and of cellular functions that, if perturbed, lead to deviations from these trends.

We evolved the selected strains for tetracycline resistance and first identified general patterns that can explain the extent to which gene-deletion strains evolved. A common pattern in evolution experiments is that identical beneficial mutations have weaker effects on fitter than on less fit backgrounds^32,33^. Here, we observed such diminishing-returns epistasis in the context of drug resistance: strains that were initially more sensitive underwent greater resistance increases during the experiment and effectively caught up with initially more resistant strains (Fig. 2b). Gene deletions often alter antibiotic resistance^35,44^; for tetracycline, the initial resistance levels of deletion strains varied by an order of magnitude (Fig. 2b). However, after 180 h of evolution (about 100 generations), these differences had largely evened out: strains with an *x*-fold lower initial resistance (IC_50_) than the wild type tended to increase their resistance by *x*-fold more in the evolution experiment (Fig. 2b). This trend is quantitatively demonstrated by a clear anti-correlation between initial resistance and fold increase in resistance (Spearman’s *ρ* = −0.544, *p* = 2 × 10^−7^); bias estimation by bootstrapping indicated that this anti-correlation is not due to a bias in our selection of gene-deletion strains (Fig. 2b and Methods). Notably, a recent study on resistance evolvability using fewer mutants from a long-term evolution experiment did not detect this diminishing-returns trend^57^ (see Discussion). As the highest resistance levels in our experiment remain ~1000-fold below the solubility limit of the antibiotic, this global pattern supports that there is a hard upper bound for spontaneous tetracycline resistance (“resistance limit”, Fig. 2b), which diminishes the possible resistance increases when approached.

### Perturbations of drug efflux pumps lower resistance evolvability

Can cells be forced to deviate from this general trend of seemingly inevitable resistance? In *E. coli* lab strains like those used in our experiments, which do not carry horizontally transferred tetracycline resistance genes such as *tetA* and *tetB*^22,58^, many tetracycline resistance mutations directly relate to the overexpression of endogenous drug efflux pumps^52,53^. Thus, we reasoned that perturbing the composition or regulation of these pumps may affect resistance evolvability. Indeed, compromising efflux pumps sensitizes bacteria to various drugs^35,59^, but the effect on evolvability is unknown. On the one hand, strains with perturbed efflux pumps are farther away from the resistance limit and thus, according to the diminishing-returns pattern (Fig. 2b), should undergo greater relative resistance increases. On the other hand, disrupting efflux pumps could effectively block mutational paths to resistance and force evolution to seek a different—likely less accessible and less beneficial—path. The latter scenario would be equivalent to a shift of the “resistance limit” in Fig. 2b to lower values. To discriminate between these two scenarios, we tested how perturbations of efflux pumps affect resistance evolvability.

**Fig. 3:**
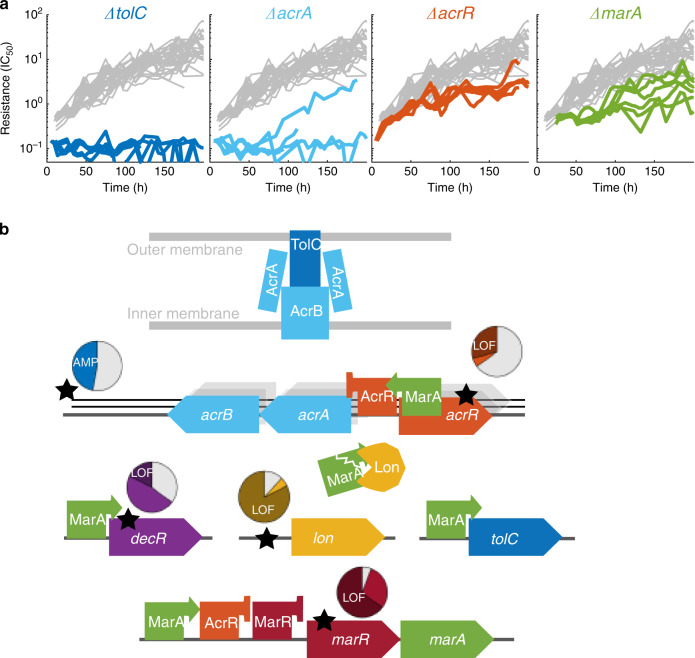
Perturbing multidrug efflux pumps can drastically reduce the evolvability of tetracycline resistance. **a** Resistance (IC_50_) over time for evolution experiments started from the *ΔtolC, ΔacrA, ΔacrR, ΔmarA* strains. All these genes are directly involved in the most common path to resistance shown in (**b**). Gray lines show resistance over time for 22 replicates of the reference strain (*ΔlacA*) for comparison. Data are from experiments M2 and M3 (Methods, Supplementary Table 2, Source data). **b** Schematic of AcrAB-TolC multidrug efflux pump in the membrane and genes that regulate it. Big arrows are genes, squares with pointing or blunt arrows are transcriptional activators or repressors, respectively, and the Pacman shape is a protease (Lon). The five most common mutation loci (Supplementary Fig. 2, Supplementary Data 1) are marked by stars; amplification of a genome region is shown as stacked black lines representing copies of DNA. Each mutation locus has a pie chart near it where colored segments represent the proportion of evolved populations started from the reference strain (*ΔlacA*) which gained a mutation in that locus during the experiment. Slices of a darker shade of the gene color represent the proportion of samples with predicted loss-of-function mutations in that locus.

We found that deleting genes that code for components of the AcrAB-TolC efflux pump can essentially block resistance evolution (Fig. 3a). The most drastic effect occurred for *ΔtolC* in tetracycline, where we detected no increase in resistance in all seven replicates of the evolution experiment (Fig. 3a). This is notable because not only did this strain evolve under the same strong selection pressure for drug resistance at the same population size for the same number of generations as all other strains, but it was even five times more sensitive at the beginning of the evolution experiment (Fig. 3a, Supplementary Fig. 6)^60^, and thus expected to increase in resistance dramatically. We detected only one fixed mutation (a single base-pair substitution in the promoter of the *yhdJ* gene) in a single *ΔtolC* evolution replicate, corroborating the lack of adaptation; this idiosyncratic mutation seemed random and was unrelated to resistance (Supplementary Data 1). A likely mechanistic explanation for the lack of adaptation in the *ΔtolC* strain is that the disruption of AcrAB-TolC effectively neutralizes the beneficial effects of resistance mutations related to this efflux pump.

Similar to *ΔtolC*, only a single *ΔacrA* replicate out of five evolved tetracycline resistance, namely by overexpressing the homologous, rarely used AcrEF-TolC efflux pump, thus circumventing *acrA* loss^61,62^. The rapidity at which evolution found this alternative mutational path highlights the difficulty of perturbing resistance evolvability. The lack of resistance evolution for *ΔtolC* (Fig. 3a) likely reflects that TolC serves as an outer membrane channel for at least eight different efflux pumps^63^, which can be disabled simultaneously. As a result, the alternative path to resistance followed in the *ΔacrA* strain is not available when *tolC* is deleted. In sum, these results exemplify the importance of detecting cellular functions that not only sensitize cells to a drug, but simultaneously hinder resistance evolution in ways that are not easily circumvented by alternative mutational paths to resistance.

We hypothesized that interfering with the regulation of efflux pumps while preserving their structural integrity provides additional ways to manipulate resistance evolvability. Indeed, several genetic perturbations of efflux-pump regulation significantly altered the rate of resistance evolution. Deleting *marA*, coding for a key activator of efflux-pump expression^45,64^ slightly increased the initial sensitivity to tetracycline and slowed subsequent resistance evolution, even if not completely abolishing it (Fig. 3a). In contrast, deleting *marR*, coding for a repressor of *marA*^65^ and therefore an indirect repressor of efflux-pump expression, increased initial resistance but had no lasting effect on resistance (Fig. 2c), following the diminishing-returns pattern. This is expected, since loss-of-function mutations in *marR* are extremely common in the experiment (Fig. 3b and Supplementary Fig. 3), and deletion of *marR* does not interfere with the usual resistance path, but rather represents a step along it, leading to the general resistance limit (Fig. 2b). Deleting *acrR*, coding for a repressor of the *acrAB* operon and the *mar* regulon^66^, increased initial sensitivity to tetracycline, likely because its coding region contains a MarA biding site that activates the *acrAB* operon^67^. Despite this greater initial sensitivity, resistance of the *ΔacrR* strain increased only modestly during the evolution experiment, by about 15-fold compared with 25-fold in the *ΔlacA* reference strain. Thus, deleting *acrR* is another way to decrease *acrAB* expression and thus limit the attainable resistance. Taken together, we identified multiple cellular targets that slow spontaneous resistance evolution by affecting efflux pumps (Fig. 3a), highlighting that even a single main mutational path to drug resistance can be targeted in several ways.

### Identification of genes that alter resistance evolvability

**Fig. 4:**
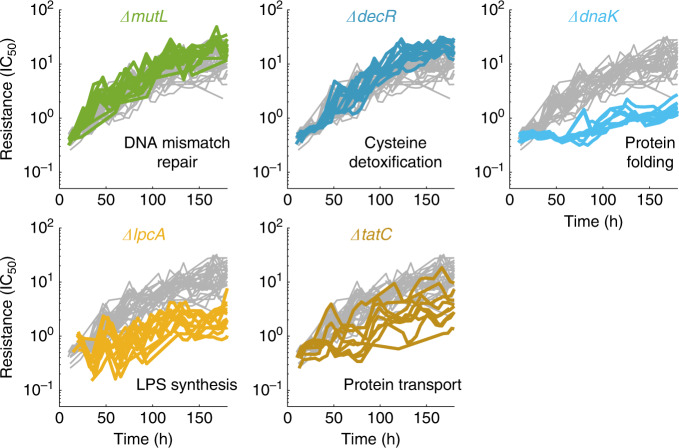
Diverse cellular functions, including chaperones, LPS biosynthesis, and DNA repair, affect resistance evolution. Resistance (IC_50_) over time as in Fig. 3a for several strains with deletions of genes that have an effect on resistance evolvability, but are not immediately related to efflux pumps. *ΔmutL* and *ΔdecR* show slightly increased evolvability, *ΔmutL* presumably due to the increased mutation rate. *ΔdnaK*, *ΔlpcA*, and *ΔtatC*, which are mutants in protein folding, LPS biosynthesis and protein export, respectively, show decreased evolvability. Gray lines show resistance over time for 22 replicates of the reference strain (*ΔlacA*) for comparison. Data are from experiments M2 and M3 (Methods, Supplementary Table 2, Source data).

Beyond efflux pumps, we revealed additional cellular functions that slow resistance evolution when perturbed. In particular, even though deleting the Hsp70 chaperone DnaK^68^ does not affect drug sensitivity, it slowed resistance evolution by almost an order of magnitude (Fig. 4). Furthermore, the *ΔdnaJ* strain which harbors a deletion of DnaK’s co-chaperone also slowed resistance evolution, albeit less extreme than *ΔdnaK* (Supplementary Fig. 7). Both observations are consistent with the notion that chaperones play a key role in evolution by affecting the conversion of genetic to phenotypic variability^69,70^. Perturbing different steps of the lipopolysaccharide (LPS) biosynthesis pathway (*ΔlpcA* and *ΔlpxM*) led to over 4- and 2-fold lower levels of final resistance, respectively (Fig. 4 and Supplementary Fig. 7). Deletion of *tatC*, a gene involved in protein transport across the membrane, also significantly slowed resistance evolution (Fig. 4). This effect is possibly due to a lower mutation rate as this gene is thought to play a role in stress-induced mutagenesis^71^. The molecular mechanisms underlying the effects of these genes on resistance evolution are unclear, highlighting the difficulty of predicting such evolvability modifiers and the importance of our systematic approach to expose them. Together, these results support that multiple independent cellular functions determine resistance evolvability; perturbing these functions often defers resistance.

Fewer of the gene deletions we investigated accelerate resistance evolution. Specifically, deleting *decR*, a regulator of cysteine detoxification^72^, slightly accelerated resistance evolution even though it did not affect initial resistance (Fig. 4). We observed that a loss-of-function mutation in *decR* occurred reproducibly in several evolved populations including the evolved reference strain (Fig. 3b, Supplementary Fig. 3a, Supplementary Data 1). This is intriguing since the *decR* deletion alone does not increase resistance and suggests that loss of *decR* function amplifies the effects of spontaneous resistance mutations. A more established way of accelerating resistance evolution are perturbations of DNA repair (*ΔmutL* in Fig. 4), which lead to mutator phenotypes with ~100-fold increases in mutation rate^47^. However, the effect was weak, suggesting that the supply rate of beneficial mutations, which should be drastically increased in the *ΔmutL* strain, is not rate-limiting for resistance evolution under our conditions. This observation indicates that resistance evolution in larger bacterial populations, such as those in our experiments, is in a regime that is not limited by the arrival of new beneficial mutations. Overall, our results suggest that bacteria are more easily perturbed in ways that slow resistance evolution rather than accelerate it. Since we tested only a few percent of the genes in the genome, this indicates a huge unexploited reservoir of candidate targets for choking spontaneous resistance evolution.

### Alterations of evolvability for different antibiotics are correlated

**Fig. 5:**
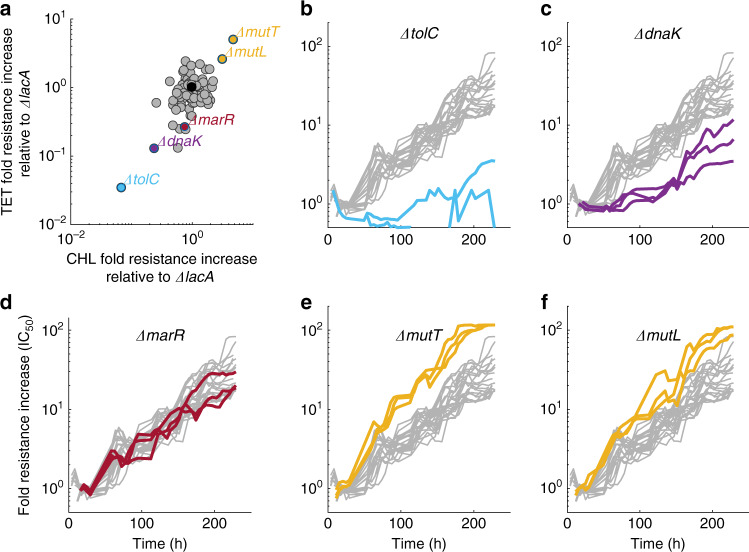
Changes in resistance evolvability are correlated across different antibiotics. **a** Fold increases in resistance to tetracycline (*y*-axis) and chloramphenicol (*x*-axis) of individual gene deletions after 180 h of evolution divided by the mean fold increase in resistance for the reference strain (*ΔlacA*) (shown in black). The relative fold increases are correlated (Pearson’s *r* = 0.63, *p* < 10^−8^). **b**–**f** Examples of resistance increase over time for chloramphenicol (CHL) for the strains highlighted in (**a**). The apparent plateauing of resistance for *ΔmutT* occurred because the concentration needed to keep the strains inhibited reached the maximum stock concentration used on that day. Gray lines show resistance over time for 23 replicates of the reference strain (*ΔlacA*) for comparison. Data are from experiment M4 (Methods, Supplementary Table 2, Source data).

To test if our results are specific to tetracycline or perhaps more general, we performed a similar evolution experiment with chloramphenicol. Like tetracycline, chloramphenicol targets the ribosome but the details of this interaction differ considerably^73^. Whereas the evolution of tetracycline resistance seemed to level off within 7 days, for chloramphenicol, we observed a steady increase even after 10 days (Fig. 5 and Supplementary Fig. 8), confirming previous reports^40^. Many mutations that fixed during the experiment overlapped with those observed for tetracycline, with additional mutations related to the MdfA efflux pump (Supplementary Fig. 3b) as previously described^40,44^. Despite these differences, the effects of specific gene deletions on evolution in the two drugs were similar, as evidenced by highly correlated increases in resistance (Pearson’s correlation coefficient *r* = 0.63, *p* < 10^−8^ for the fold-change in resistance at the end of the evolution experiment, relative to the fold-change in the *ΔlacA* strain; Fig. 5a). In particular, the perturbations with the strongest effects were common to both antibiotics: mutator strains (*ΔmutT* and *ΔmutL*) adapted faster while the *ΔtolC*, *ΔdnaK*, and *ΔmarR* strains adapted more slowly (Fig. 5b–f). The accelerated evolution in mutator strains was clearer for chloramphenicol (Fig. 5e, f). These results show that the cellular functions we identified do not just affect evolvability for one specific antibiotic in an idiosyncratic way. While it is not clear that the same genes will affect resistance evolvability for other antibiotics, these results suggest that hitting the same target can often modify resistance evolvability for at least several different drugs.

### Epistatic interactions underlie evolvability alterations

**Fig. 6:**
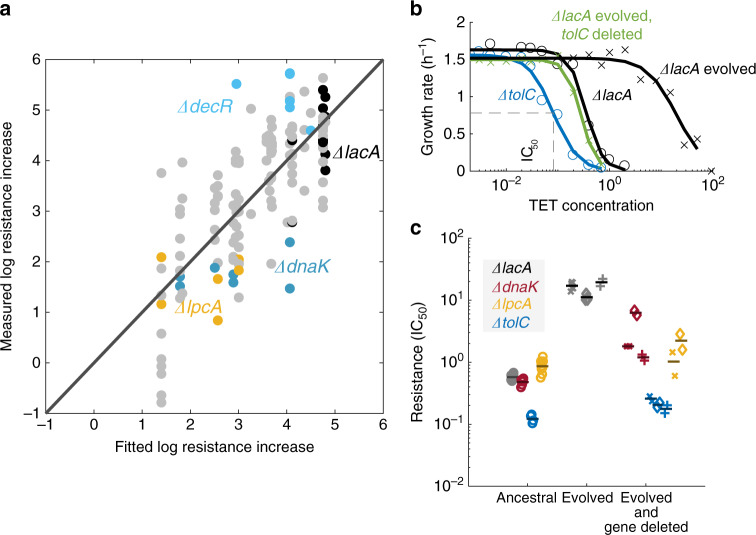
Epistasis between gene deletions and resistance mutations underlies altered evolutionary dynamics. **a** Measured vs. predicted log resistance increase for each sequenced evolved population. For the prediction, the five most common mutations were considered (Methods). The black circles represent control (*ΔlacA*) strains. *ΔdecR*, *ΔlpcA*, and *ΔdnaK* samples are highlighted to visualize that their effects systematically deviate from predictions. **b** Dose–response curves for four strains. Black circles represent the ancestral *ΔlacA*, blue circles represent the ancestral *ΔtolC*, black crosses represent a clone isolated from an evolved *ΔlacA* population and green crosses represent the same clone with *tolC* deleted. A Hill function is fitted to each set of measurements. The value of the IC_50_—the concentration at which the growth rate is half of the maximum—is shown with dashed gray lines for *ΔtolC*. **c** Resistance level (IC_50_) of the ancestral strains (circles): *ΔlacA* (reference, gray, *n* = 32), *ΔdnaK* (red, *n* = 8)*, ΔtolC* (blue, *n* = 4), and *ΔlpcA* (yellow, *n* = 9); three different resistant (evolved from *ΔlacA*) clones (crosses, *n* = 6, diamonds, *n* = 6, plus signs, *n* = 2), and strains where *dnaK*, *tolC*, and *lpcA* are deleted on the background of the respective resistant clones (*n* = 2, Methods). Each symbol shows an IC_50_ determined from an independent measurement of the dose–response curve (as shown in panel **b** for *ΔtolC*); for all ancestral strains, the IC_50_ was determined independently from the beginning of experiments M2 and M3 as in Fig. 2b (Methods). Black horizontal lines represent the mean resistance value of each strain. The deletions of each gene (*dnaK, tolC,* and *lpcA*) on the resistant background significantly decrease resistance (left to right: *p* = 7 × 10^−7^, *p* = 5 × 10^−4^, *p* = 4 × 10^−3^, *p* = 3 × 10^−8^, *p* = 7 × 10^−9^, *p* = 2 × 10^−3^, *p* = 2 × 10^−5^, *p* = 4 × 10^−5^, two-sided *t*-test on log values, Methods). The resistance decrease due to these deletions on the resistant background is greater than on the sensitive background, exposing epistasis between resistance mutations and these gene deletions (left to right: *p* = 7 × 10^−9^, *p* = 2 × 10^−3^, *p* = 3 × 10^−9^, *p* = 3 × 10^−5^, *p* = 4 × 10^−5^, *p* = 3 × 10^−5^, *p* = 3 × 10^−7^, *p* = 5 × 10^−6^, two-sided *t*-test on normalized values, Methods). Deleting *tolC* in the evolved strains lowers their resistance to slightly below the initial resistance level of the reference strain. The evolved strains used are lacA-M3-1–3 (crosses), lacA-M3-2–2 (diamonds), and lacA-M3-2–3 (plus signs); see Supplementary Data 1 for detailed information on the mutations that occurred in the evolved strains.

We hypothesized that many of the observed changes in evolvability are caused by epistasis between the gene deletions and common spontaneous resistance mutations. To test this hypothesis, we first combined our whole-genome sequencing data for evolved strains with the resistance levels measured at the end of the evolution experiment. Based on these data, we built a simple linear regression model to estimate the benefit of each spontaneous resistance mutation (Methods). This model enabled us to identify deletion strains where these mutations fixed but had a different resistance benefit than expected (Fig. 6a). This analysis indicated magnitude epistasis, i.e., a quantitative change in the fitness effect of a mutation due to the presence of a different mutation^31^, between the deletion and the acquired mutations. For example, in a *ΔdnaK* background, the same resistance mutations increased resistance by considerably less than in other strains (Fig. 6a). In extreme cases where the gene deletion completely nullifies the benefit of spontaneous resistance mutations, these mutations would not fix in the evolution experiment, as observed for the *ΔtolC* strain (Fig. 3a, Supplementary Data 1). Thus, the strongest epistatic effects are not detectable by this approach.

To extend this analysis to extreme cases like *ΔtolC* and corroborate the central role of epistasis in resistance evolvability, we directly quantified epistatic interactions between specific gene deletions and common resistance mutations. We isolated clones from the *ΔlacA* reference populations evolved in tetracycline and deleted genes that modified the rate of resistance evolution in our experiments. We then measured the IC_50_ of the ancestral, evolved, and newly modified strains (Fig. 6b, Methods). Deleting *tolC* rendered the evolved reference strains even more sensitive to tetracycline than the ancestral reference strain (Fig. 6b, c). Deletion of *dnaK* or *lpcA* also sensitized the resistant strains, albeit only partially (Fig. 6c). These results expose epistatic interactions between resistance mutations and gene deletions identified in our large-scale search for evolvability modifiers, which are consistent with their slower resistance evolution. More quantitatively, the extent of epistasis mirrored the observed differences in resistance evolvability for *ΔtolC* and *ΔdnaK*, respectively (Figs. 3 and 4). There is a plausible mechanism for the epistatic interactions between *ΔtolC* and the common resistance mutations: since many of the resistance mutations affect the AcrAB-TolC multidrug efflux pump, which is likely disabled by *tolC* deletion, these mutations lose their beneficial effects. Together, these results highlight the potential of exploiting epistatic interactions to restrain spontaneous resistance evolution.

## Discussion

We presented a systematic analysis of the effects of targeted genetic perturbations on spontaneous antibiotic resistance evolution in well-controlled laboratory experiments. We identified a general pattern of diminishing-returns epistasis that guides resistance evolution (Fig. 2). Perturbations of specific cellular functions clearly deviate from this trend (Figs. 3 and 4) and affect resistance evolvability most drastically due to strong epistatic interactions with resistance mutations (Fig. 6). To obtain these results, we established an automated, high-throughput experimental evolution platform that keeps hundreds of cultures in parallel in exponential phase under controlled selection pressure (Fig. 1). This platform allows precise detection of adaptation rates over a wide dynamic range and is broadly applicable. It enables quantitative investigations of evolvability for diverse microbes and other stressors than antibiotics.

The diminishing-returns trend we observed for antibiotic resistance extends similar observations of global epistasis that were made at the level of fitness or growth^32,74^. In essence, we found a strong tendency that gene-deletion mutants converge on a fixed resistance limit by accumulating resistance mutations under drug selection, irrespective of their initial resistance: The effects of resistance mutations become weaker in more resistant genetic backgrounds (Fig. 2b). This indicates that loss-of-function mutations transform the fitness landscape of antibiotic resistance in a largely predictable fashion. However, a recent study that investigated resistance evolvability for clones derived from a long-term evolution experiment detected no signs of diminishing-returns epistasis for tetracycline and other antibiotics, and concluded that resistance evolvability is historically contingent on prior mutations^57^. These diverging conclusions might be due to the different *E. coli* strain backgrounds and mutants used: Card et al.^57^ investigated four clones carrying multiple mutations that fixed under the selection conditions in the long-term evolution experiment, while we studied a diverse set of defined gene-deletion strains. Detecting the diminishing-returns trend is facilitated by the increased statistical power of our approach, resulting from the investigation of almost 100 lines, and the considerably greater fold increases in resistance that occur in our assays, which select multiple resistance mutations over more than a week while dynamically adjusting drug concentration to maintain selection pressure, as also discussed in ref. ^57^. Future studies extending this work to other antibiotics and bacteria are needed to resolve if diminishing-returns epistasis is a general phenomenon in antibiotic resistance.

We provided a systematic way of identifying genes that drastically slow resistance evolution when deleted; specific examples are *tolC*, *dnaK*, and *lpcA* (Figs. 3 and 4). In principle, the products of such genes could be candidate drug targets for a strategy in which antibiotics are combined with compounds that slow down the evolution of resistance without any need for lowering the mutation rate. The most notable candidate identified here is *tolC*: its deletion not only impairs spontaneous resistance evolution, which we showed here, but also sensitizes the cell to many antibiotics^60,75,76^. A TolC-inhibitor would strongly synergize with antibiotics like tetracycline and chloramphenicol^60^ while at the same time slowing resistance evolution. Thus, in evolution experiments, this strategy may circumvent the general trend that synergistic drug combinations tend to accelerate resistance evolution^77^. In clinical isolates, the prominence of horizontally transferred resistance genes limits direct applications for the specific antibiotics we focused on here. Notably, however, a recent study showed that acquisition of resistance genes like *tetA* by plasmid transfer in the presence of tetracycline depends on the AcrAB-TolC multidrug efflux pump, which reduces the intracellular tetracycline concentration and thus enables TetA translation^78^. Consequently, a TolC-inhibitor could hamper the horizontal transfer of resistance genes while simultaneously slowing the evolution of spontaneous tetracycline resistance, which possibly becomes more relevant when horizontally transferred resistance is unavailable.

To follow this potential strategy, small molecules targeting the evolvability modifiers identified using our approach are needed. For this purpose, one could test antisense oligomers (phosphorodiamidate morpholino oligomers, PPMOs), which are a promising way to inhibit the expression of a broad range of targets^79^—an approach that has been successfully used for genes encoding efflux pumps, leading to antibiotic hyper-sensitivity^60^. Targeting efflux pumps would also be possible with other small molecules^80–83^ or with phages that require TolC for entry^84^. This specific approach has broader potential for gram-negative bacteria, where TolC family proteins are ubiquitous^85^. As a general strategy, discovering inhibitors of evolvability modifiers along with inhibitors of horizontally transferred resistance could reinvigorate old drugs and, at the same time, put their use on a more sustainable future trajectory.

There is a plausible molecular mechanism for the negative epistatic interactions between the *tolC* deletion and the common resistance mutations (Fig. 6b, c), which drastically lower resistance evolvability: the *tolC* deletion effectively disables AcrAB-TolC and other efflux pumps that use TolC as their outer membrane channel. As a result, the usual resistance mutations, many of which affect the function and expression of these efflux pumps (Fig. 3b), are effectively neutralized. While epistatic interactions with common resistance mutations also contribute to the altered evolutionary dynamics caused by gene deletions such as *ΔdnaK, ΔlpcA, ΔtatC*, and *ΔdecR* (Fig. 6), the underlying molecular mechanisms of these epistatic interactions remain to be further studied. Chaperones such as DnaK were proposed to affect evolution by buffering the phenotypic effects of mutations^54,70,86^. There is also evidence that chaperones may enhance the phenotypic effects of spontaneous mutations^87^. The latter scenario is consistent with our observations: If DnaK amplified the effects of spontaneous mutations, its deletion would weaken the beneficial effects of spontaneous resistance mutations and slow the rate of resistance evolution as observed in our assay (Figs. 4 and 5c). However, in the case of efflux-pump related resistance, an alternative explanation is that TolC is a predicted client of DnaK^88^—hence, the main effect of the *dnaK* deletion could be that it effectively knocks down *tolC*, which in turn affects resistance evolvability by the mechanism described above.

Similarly, perturbing membrane composition via LPS biosynthesis as in the *ΔlpcA* strain might slow resistance evolution (Fig. 4) by interfering with the proper function of efflux pumps, which would again weaken the effects of the most common resistance mutations. However, there is evidence that perturbing the assembled LPS layer itself does not change efflux-pump activity^89^. The mechanisms underlying the faster adaptation of the *ΔdecR* strain (Fig. 4) and the frequent occurrence of spontaneous mutations in this locus remain to be elucidated. DecR was recently shown to be a repressor of a single operon that is involved in L-cysteine detoxification^72^; *decR* is upregulated by MarA and TolC is involved in L-cysteine transport^90^. Therefore, the resistance mutations affecting these genes may also affect DecR and L-cysteine levels, introducing a cost or sensitivity, which could in turn be alleviated by specific mutations in this regulator. In general, elucidating the molecular mechanisms underlying the epistatic interactions that alter resistance evolvability is challenging, not least because these mechanisms can be different in each individual case. Still, this problem needs to be addressed in future work.

Technological advances may enable investigating the effects of all ~4000 viable *E. coli* gene deletions^41^ (and other mutant collections) on resistance evolvability in the future. This approach could provide a comprehensive overview of the role of all genes in resistance evolvability, analogous to studies that characterized the phenotypic effects of all genes on growth in the presence of antibiotics and other chemicals^35,44^. Still, even if the rigorous feedback-control of population size and selection pressure we used here is relaxed to simplify the experiment, following this route will require considerable advances in lab automation. For global results, such as the diminishing-returns trend (Fig. 2b), it often suffices to investigate a few percent of the genome; this trend would almost certainly be confirmed in a genome-wide investigation. Overall, our study provides a stepping-stone from typical investigations of resistance evolution in a single or a few different genotypes toward systematic investigations of genome-wide perturbations on evolvability, which may become feasible in the future.

The ability to perform an even greater number of evolution experiments would also facilitate a systematic investigation of resistance evolvability for a larger set of different antibiotics representing all major modes of action. This approach would clarify if the cellular functions and specific genes we identified as modifiers of resistance evolvability are specific for tetracycline and chloramphenicol or more general across different antibiotic classes. Even if different cellular functions need to be targeted to modify resistance evolvability for other antibiotics, our work provides a systematic way of exposing these functions. Importantly, while targeting the AcrAB-TolC multidrug efflux pump is only expected to slow resistance evolution for antibiotics that are transported out of the cell by this pump, the concept of targeting known resistance mechanisms to slow resistance evolution is likely more general. We restricted the present study to two ribosome inhibitors because many antibiotics with other modes of action pose additional challenges for precise feedback-control of population size and selection pressure. For example, quinolones act with time delays of several hours, while beta-lactams have highly sensitive dose–response characteristics^44^. Hence, investigating other antibiotic classes, while feasible, requires careful adaptation of our automated evolution platform. Nevertheless, extending our approach to other antibiotic classes is a promising direction of future research.

Even when resistance-enabling genes like *tolC* or *dnaK* are disabled and horizontal gene transfer is prevented, evolution would ultimately find ways to increase resistance, but our results indicate that this could take orders of magnitude longer. A potential strategy for slowing resistance evolution even further would be to combine an antibiotic with inhibitors for several of the key resistance-enabling genes identified using the approach presented here. In this way, even less-common paths to resistance could be blocked and the probability of circumventing these blocks by mutation might become prohibitively low. This work shows that most gene deletions affect the fitness landscape of antibiotic resistance in a predictable way and provides a framework for identifying perturbations that fundamentally alter the local properties of this landscape. It will be interesting to extend this approach to other organisms and drugs.

## Methods

### Strains, media, reagents, and antibiotics

Cultures were grown in LB medium from Sigma-Aldrich (#L3022). For PCR reactions GoTaq G2 DNA Polymerase (Promega #M7845) or Q5 high fidelity Polymerase (New England Biolabs #M0491S) were used.

All strains originated from isolated clones (plated on solid LB, picked, regrown overnight in LB and frozen in 15% glycerol) from the Keio collection^41^ with kanamycin cassette included in the locus of the deleted gene.

Tetracycline stock solutions of 7 or 10 mg/ml were prepared by diluting tetracycline hydrochloride powder (Sigma-Aldrich # T7660) in 83% ethanol at room temperature. Chloramphenicol stocks of 10 mg/ml were prepared by diluting powder (Sigma-Aldrich #C0378) in 99% ethanol. Kanamycin stock was made from kanamycin-sulfate powder (Sigma-Aldrich #K4000). All antibiotic stocks were stored at −20 °C.

### Whole-genome sequencing analysis

Whole-genome sequencing was performed for 380 samples altogether as listed in Supplementary Data 1. For all evolved population samples, the ancestral clone was also sequenced and its mutations analyzed (Supplementary Data 1), to distinguish clearly between mutations acquired before and during the experiment. Genomic DNA was purified directly from thawed glycerol stocks using the GenElute 96 Well Tissue Genomic DNA Purification Kit (Sigma-Aldrich # G1N9604). Library preparation, multiplexing, and sequencing were performed by LGC Genomics GmbH. The samples were sequenced on an Illumina NextSeq500 V2 (paired-end sequencing, 150 bp read length, ~230-fold coverage on average, but ranging from ~70- to ~800-fold due to the multiplexing protocol). Sequencing data were analyzed using Breseq^91^ (version 0.32.0). Reads were aligned to the deposited Keio parent reference (Accession: CP009273) using Bowtie (version 1.2.11). The mutations identified by Breseq were manually inspected for false positives; all validated mutations are listed in Supplementary Data 1. Even though the samples were expected to be heterogeneous (they were not isolated clones), the “clonal” mode of Breseq was used. Therefore, the mutations detected only represent fixed mutations. Amplifications were noted if the coverage of a multi-genic region (which includes the *acrAB* or *mdfA* operon and is over 5000 bp) exceeded twice the median coverage of that sample. Since an IS insertion in the *lon* promoter region was often among the “unassigned new junction evidence” but at very high frequency, this type of mutation was assumed to be fixed if the frequency exceeded 90%. For each evolved sample, we validated that the intended gene deletion is present. If any reads in the deletion locus were present, which would suggest cross-contamination with another strain, the sample was excluded from the analysis.

### Automated experimental evolution

The selected deletion strains from the Keio collection^41^ as listed in Supplementary Table 1 were all streaked for single colonies and clonal cultures frozen with 15% glycerol at −80 °C. The glycerol stocks were used to assemble the starting 96-well plates for the evolution experiment. Each plate had at least 12 empty wells, which were filled with sterile growth medium, and handled like all other wells throughout the experiment to monitor cross-contamination. Replicates of the same ancestor, if on the same plate, were placed far from each other to avoid cross-contamination which would not be detected by genotyping. Every plate contained at least two replicates (and usually more) of the control strain (either the BW25113 parent strain of the Keio collection or the *ΔlacA* strain). The *ΔlacA* strain was used as reference in later experiments instead of the BW25113 to ensure that any difference between the strains is not due to the presence or absence of the kanamycin cassette.

The automated evolution protocol was carried out four times using a Tecan Freedom Evo 150 liquid-handling platform. The specific differences between the runs of the experiment are given in Supplementary Table 2. The 200 µl cultures were kept in LB rich medium in 96-well plates (Nunc, transparent flat-bottom) in a shaking incubator (Liconic Storex, 30 °C, >95% humidity, 720 rpm). Continual shaking and rich growth medium ensured that the cultures were homogeneous and not under oxygen or nutrient limitation. Every 10–15 min, each plate was transferred to a plate reader (Tecan Infinite F500) using a robotic manipulator arm (RoMa) and the absorbance (OD at 600 nm) was measured. Every 3–5 h, the cultures were transferred to new plates. They were not diluted in the same plates to avoid biofilm formation and due to large errors in volumes left in the wells after pipetting out most of the culture. The new plate was filled in three steps. First, pure LB medium (*v*_med_) was pipetted, then medium with antibiotic (*v*_ab_) and last the culture (*v*_culture_) from the previous plate. Each culture had its own dedicated 200 µl disposable tip, which was washed in ethanol after every dilution. LB medium and antibiotic stock were multi-pipetted into the new plates using 1000 µl tips. All tips were exchanged once a day. The reservoirs with media had lids that were taken off using the RoMa arm just before usage.

Every day of the experiment, the penultimate plates of that day were left in the incubator to grow out: the next day 70 µl of 50% glycerol was added to each well and the plates frozen at −80 °C. Fresh antibiotic stocks and medium reservoirs were provided. There were always two concentrations of antibiotic stocks available, ~10-fold apart, the protocol always only chose one of the available stocks to pipette from. The concentrations of the antibiotic stocks were chosen each day depending on how resistant the populations had become.

Every 3–5 h, the cultures from each plate were transferred to new plates using the Air LiHa robotic pipetting head. The appropriate volumes of culture, medium and antibiotic to use were calculated at each dilution step and for each culture using a custom Python script based on the OD values obtained since the last dilution. The growth rate was obtained from 18 consecutive OD measurements by obtaining the slope of the least-squares linear fit (numpy.polyfit function) to the log_2_ of those background subtracted OD values which were between 0.01 and 0.1. All growth rates were normalized to the growth rate of the reference strain in the absence of antibiotic (1.7 doublings per hour). The volumes were calculated separately for each well using the last OD measurement (*d*), normalized fitted growth rate (*g*_current_), concentration of antibiotic stock (*c*_stock_), current antibiotic concentration in the well (*c*_current_) and Hill coefficient of the dose–response curve *n*_TET_ = 1.8, *n*_CHL_ = 2.4^44^ in order to reach the target OD (*d*_target_ = 0.01), growth rate (*g*_target_ = 0.5), and total volume (*v*_total_ = 200) according to the equations:

1 $$V_{{\mathrm{culture}}} = V_{{\mathrm{total}}} \cdot d_{{\mathrm{target}}}/d$$

2 $$V_{{\mathrm{culture}}} = V_{{\mathrm{total}}} \cdot d_{{\mathrm{target}}}/dc_{{\mathrm{target}}} = c_{{\mathrm{current}}}\left( {\frac{{g_{{\mathrm{current}}}}}{{1 - g_{{\mathrm{current}}}}}} \right)^{\frac{1}{n}}$$

3 $$V_{{\mathrm{ab}}} = (v_{{\mathrm{total}}} \cdot c_{{\mathrm{target}}} - b \cdot c_{{\mathrm{current}}})/c_{{\mathrm{stock}}}$$

4 $$V_{{\mathrm{med}}} = V_{{\mathrm{total}}} - V_{{\mathrm{culture}}} - V_{{\mathrm{ab}}}$$

We took several precautions to deal with atypical input values. If the concentration *c*_current_ is zero, *c*_target_ is set to a default concentration of 0.1 µg ml^−1^ for tetracycline and 0.5 µg ml^−1^ for chloramphenicol, which are values lower than the IC_50_ of the most sensitive strains in our selection. If the sum of squared residuals from the fit to obtain the growth rate is >0.8, which was empirically chosen to reflect a failed growth-rate fit, then *c*_target_ is set to *c*_current_. If the measured normalized growth rate is larger than 0.9, it is set to 0.9 to avoid very large or undefined values for *c*_target_ due to the sigmoidal shape of the dose–response curve. If the calculated volume *V*_ab_ is smaller than 5 µl, *V*_ab_ is set to zero (only medium is used to dilute the culture) and concentrations are updated accordingly. *V*_culture_ is capped at 140 µl, to assure accurate aspiration from the small 200 µl culture. There were two available reservoirs of antibiotic stocks, the higher concentration was only used if, for the lower stock concentration

5 $$V_{{\mathrm{ab}}} \, > \, V_{{\mathrm{total}}} - V_{{\mathrm{culture}}}.$$

### Resistance measure

The “on-the fly” resistance (IC_50_) measure for a particular culture is the antibiotic concentration in the well at that time. The concentration was updated at every dilution. For all plots of resistance over time (Figs. 2–4) and all “initial” and “final” resistance measures, only those time points where the growth rate after that particular dilution was close to half-inhibited (between 0.3 and 0.7 of the maximum growth rate of the reference strain) were considered.

### Correlation analysis and bootstrapping

For the diminishing-returns trend in Fig. 2b, bootstrapping analysis was performed to estimate the confidence interval and bias of the calculated correlation. To quantify the strength of the trend, we used the Spearman (rank) correlation *ρ*, which is insensitive to extreme outliers like *tolC* (Supplementary Fig. 6). Data points were resampled 10,000 times (Matlab function bootstrp) to obtain the 95% confidence interval. The mean value of *ρ* from the bootstrapped data was only 0.005 higher (−0.593) than the sample correlation (−0.598), showing that sampling bias has a negligible effect on the observed trend. For all correlations (Figs. 2b, 5a, and Supplementary Fig. 1), a permutation test was performed to obtain the *p*-value of the correlation.

### Regression model of mutational effects

The regression model has two major assumptions. First, different mutations in the same locus provide the same resistance benefit and second, the effects of mutations on resistance are additive on a log scale, i.e., each mutation brings a fixed relative resistance increase irrespective of which other mutations are present. Assuming this is true, the log resistance levels **y** can be expressed as a linear model:

6 $${\mathbf{y}} = b_0 + {\mathbf{b}} \cdot {\mathbf{x}} + {\it{\epsilon }},$$

where **y** is the log of the increase in resistance observed for the individual evolving populations, *b*_0_ is a fitted coefficient corresponding to the resistance increase common to all evolved populations not predicted by the five most common mutations, **b** is the vector of fitted coefficients which correspond to the effects of the individual mutations, **x** is a vector of ones or zeros determining the presence or absence of that particular mutation in the given evolved population.

Mutations from all sequenced samples evolved in tetracycline which passed contamination and quality control were included in the analysis Supplementary Data 1. The function fitlm (Matlab R2016b) was used. The predicting variables were the presence and absence of mutations in the five most commonly hit genes (*marR*, *lon*, efflux-pump amplification, *acrR*, *decR*), the fit parameters were the multiplicative effects of mutations in those loci, and the response variable was the log resistance increase over the course of the experiment. The predicted mutational effects of the five most common mutations are given in Supplementary Table 3.

### Generation of double-deletion mutants

All newly constructed strains (used in Fig. 6b, c) are available upon request.

Before introducing the kanamycin cassette into the evolved *ΔlacA::kan*^*R*^ strains to produce double-deletion mutants, clones were picked from LB agar plates with 25 µg ml^−1^ kanamycin sulfate and 10 µg ml^−1^ tetracycline hydrochloride and their growth rates were compared with the evolved strain. The growth rate was determined in a dose–response assay as explained in “Dose-response measurements”. Clones with resistance level similar to the evolved population were subjected to P1-phage transduction (for *dnaK* and *tolC* deletion) or lambda-red recombineering (for *lpcA* deletion).

Prior to the P1-phage transduction, the FRT-flanked kanamycin cassette has been removed from the evolved *ΔlacA* strains with the plasmid pCP20 and selection on LB agar with 100 µg ml^−1^ ampicillin and with 25 µg ml^−1^ kanamycin. Afterward the double-deletion mutants were created by transferring the respective alleles *(ΔdnaK, ΔtolC, ΔlpcA*, and Δ*lacA*) from the Keio collection into *ΔlacA* evolved strain using the standard P1-phage transduction protocol^92^. The genotype was verified after P1 transduction with PCR (Supplementary Table 4).

To delete the *lpcA* gene in the evolved *ΔlacA* strains, the chromosomal gene *lpcA* was targeted with lambda-red-mediated homologous recombination, due to inefficient P1 infection of the *ΔlpcA* strain. A PCR product containing the kanamycin cassette flanked by FLP recognition target sites and 50 base pairs homologies to adjacent chromosomal sequences^41^ (Supplementary Table 4), and 20 bp homology to the plasmid pKD13, were amplified using Q5-HF-polymerase (NEB). The PCR product was purified using a standardized PCR clean-up kit (Promega #A9282) and electroporated into evolved *E. coli* BW2511 *ΔlacA::kan*^*R*^ with the recombineering plasmid pSIM19. The transformed cells were selected for kanamycin (25 µg ml^−1^) and the presence of the PCR product was confirmed by colony PCR (Supplementary Table 4).

### Analysis of the epistasis effects of gene deletions

In Fig. 6c, the significance of the effects of gene deletions was determined by performing a two-sided *t*-test on the relative resistance change due to the gene deletion on the ancestral (sensitive) versus evolved (resistant) backgrounds. For this test, IC_50_ values of gene deletions from the Keio collection^41^ (Δ*dnaK, ΔtolC, ΔlpcA*) were normalized to the mean IC_50_ value of the reference Δ*lacA* strain; IC_50_ values of the constructed double-deletion strains (gene deletions on background of evolved Δ*lacA* strains) were normalized to the mean IC_50_ of their corresponding background resistant clones.

### Dose–response assay

Strains were grown overnight at 30 °C in LB broth without any antibiotics for 20 h. The growth rate of the double- and single-gene-deletion mutants with and without tetracycline were determined at OD_600_ using the Biotek plate reader Synergy H1. The overnight culture was diluted 1:1000 in all assays. The cell growth was observed for 25 h at 30 °C.

The Hill function fits were obtained by fitting the function

7 $$y = \frac{{g_0}}{{1 + \left( {\frac{x}{{c_0}}} \right)^n}}$$

to the growth-rate measurements using the function fit (Matlab R2016b). *g*_0_ is the fitted maximum growth rate (or the growth rate without drug), *c*_0_ is the fitted IC_50_ and *n* is the fitted dose sensitivity^44^.

### Growth-rate fits

Unless specified otherwise, growth rates are determined as the slopes of a linear fit to the log_2_ background subtracted OD values. Only those OD values which lie between 0.015 and 0.1 (after background subtraction) and only the time window from when the values first cross 0.015 until they reach 0.2 were considered. The function fit (Matlab R2016b) was used.

### Reporting summary

Further information on research design is available in the Nature Research Reporting Summary linked to this article.

## Supplementary information

Supplementary Information

Peer Review File

Description of Additional Supplementary Files

Supplementary Data 1

Reporting Summary

## Data Availability

Whole-genome sequencing data is accessible in the European Nucleotide Archive under accession code PRJEB37495. All other data are included within the main text or supplementary materials. Figures 3b, 6a, Supplementary Fig. 3 and 4 are built on the sequencing data and resulting mutation calls available in Supplementary Data 1. Data to produce all other figures are available in Source data. Source data are provided with this paper.

## Source data

Source Data

## Code availability

This study did not generate any new computer code beyond basic data analysis code, which is available from the corresponding author upon request. Source data are provided with this paper.
