# Supplementary material for: Highly parallel lab evolution reveals that epistasis can curb the evolution of antibiotic resistance
Source: Nat Commun. 2020 Jun 19;11:3105. doi: 10.1038/s41467-020-16932-z (PMC7305214; doi:10.1038/s41467-020-16932-z)
Supplement: Supplementary file 1 — Supplementary Information [file 41467_2020_16932_MOESM1_ESM.pdf]

Supplementary Information for

Highly parallel lab evolution reveals that epistasis can curb the evolution of antibiotic resistance

Lukačišinová et al.

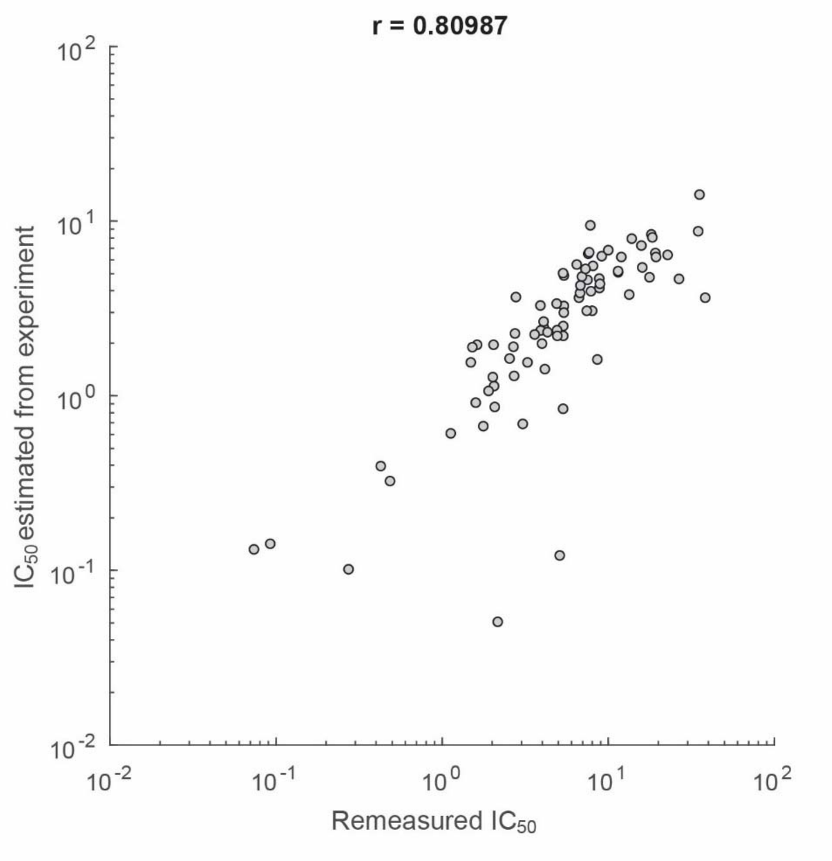

Supplementary Fig. 1 | Antibiotic concentration in well during evolution experiment agrees with conventional IC_{50} measurement.

The concentration of tetracycline in the well at the end of the experiment for many wells is plotted against the fitted IC_{50} values from measuring the growth rate of the same populations in a wide range of tetracycline concentrations (Methods). The Pearson's correlation coefficient calculated from the log values of the two measurements is given in the title of the plot ($p<10^{-10}$).

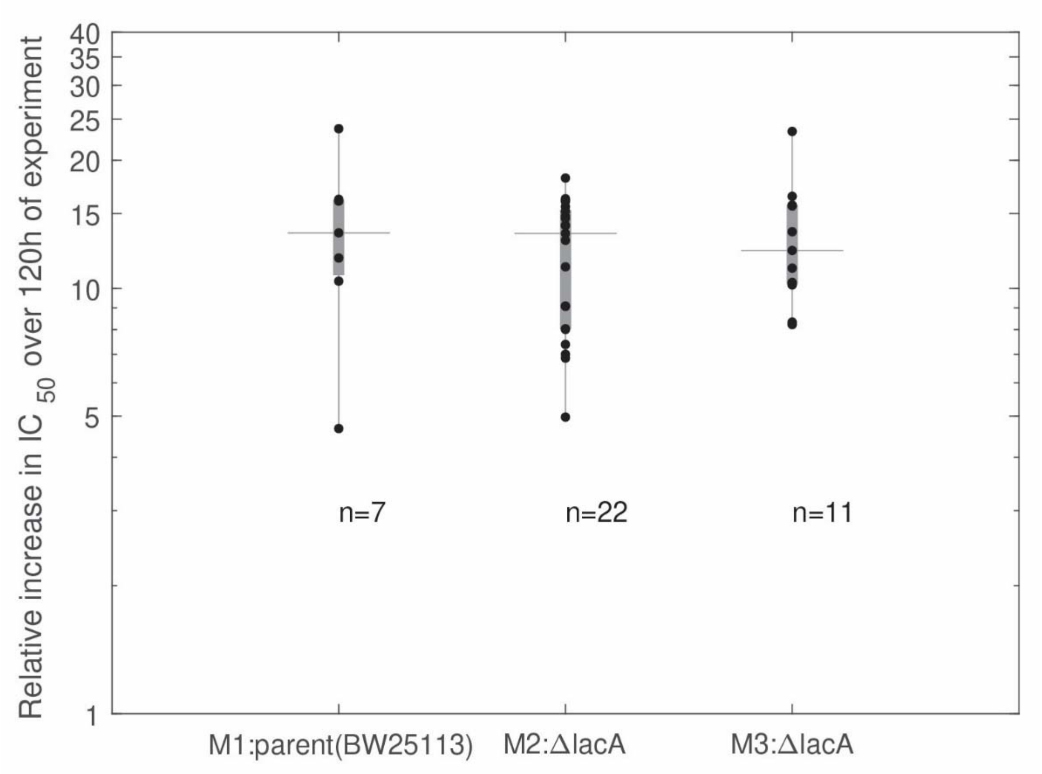

Supplementary Fig. 2 | Resistance increases for Keio parent strain (BW25113) and Δ*lacA.*

Fold resistance increases over 120h of the evolution experiment for 7 replicates of the Keio parent strain (BW25113) used in experiment M1 and 22 (M2) and 11 (M3) replicates for the Keio Δ*lacA* strain used as a reference strain in the other experiments. Individual data points as well as box plot summaries in grey are shown. The line represents the median, the box edges are the 25th and 75th percentiles and the whiskers extend to the full range of data points, meaning that there are no outliers (outside 2.7σ from the median if the data were normally distributed). Since experiment M1 lasted for 120h (and M2 and M3 for over 180h), to allow for a direct comparison, increase over 120h is reported for both strains. The differences in resistance increase are not significantly different (double sided t-test, M1 vs M2: p=0.385, M1 vs M3: p=0.822).

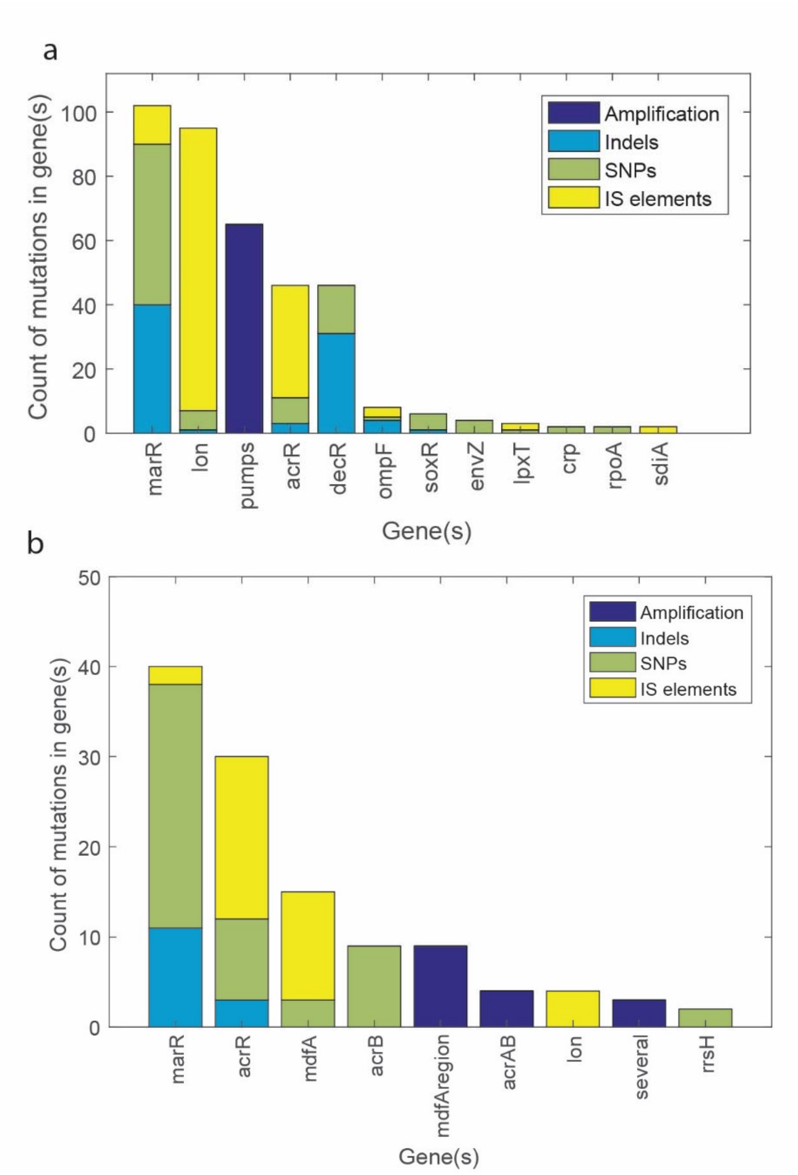

Supplementary Fig. 3 | Mutations reproducibly occur in a small number of loci during evolution in tetracycline and chloramphenicol.

a) Counts and types of fixed mutations found in n=165 independent populations evolved in tetracycline and grouped by gene locus where they occurred. Only genes which were hit at least twice in our dataset are shown. The label "pumps" denotes an amplification of the region of the *acrAB* operon identified from a more than two-fold increase in coverage compared to the median (Methods). The label "several" denotes an amplification of a different region spanning several genes. The five most common mutations represent 84% of the total mutations identified. b) The same chart as in a) for mutations identified in n=56 independent populations evolved in chloramphenicol.

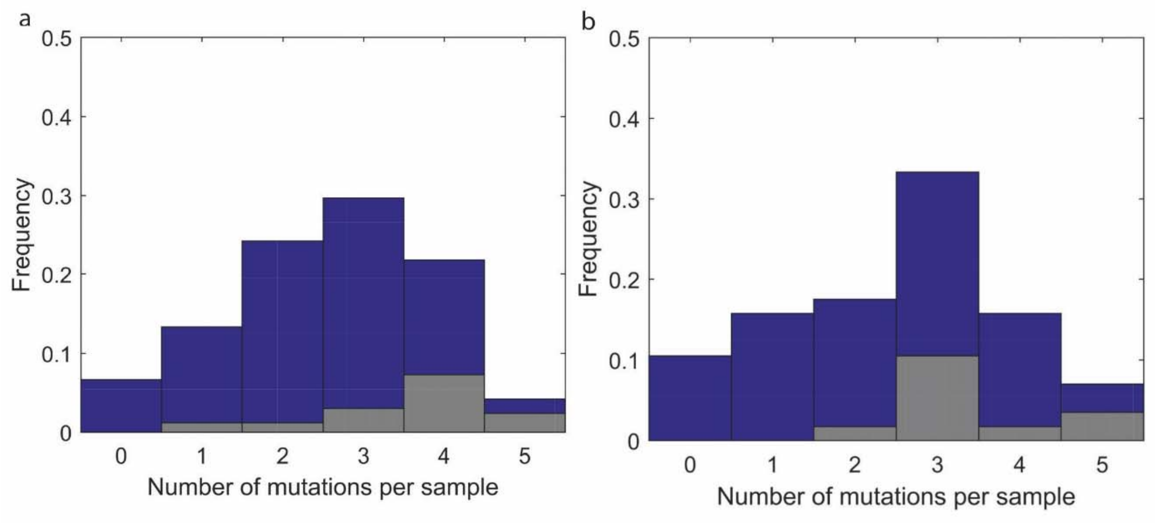

Supplementary Fig. 4 | Number of identified fixed mutations in evolved populations.

a) Histogram of the number of mutations detected in each sample (n=165) evolved in tetracycline. The gray bars represent the number of mutations found in reference strains: the Keio parent strains or the Δ*lacA* strain. The typical number of fixed mutations for reference strains is 3 or 4. Since the selection sent for sequencing was biased toward slowly evolving strains, the overall counts are biased to fewer mutations. b) The same chart as in panel a for the counts of mutations identified in populations (n=56) evolved in chloramphenicol.

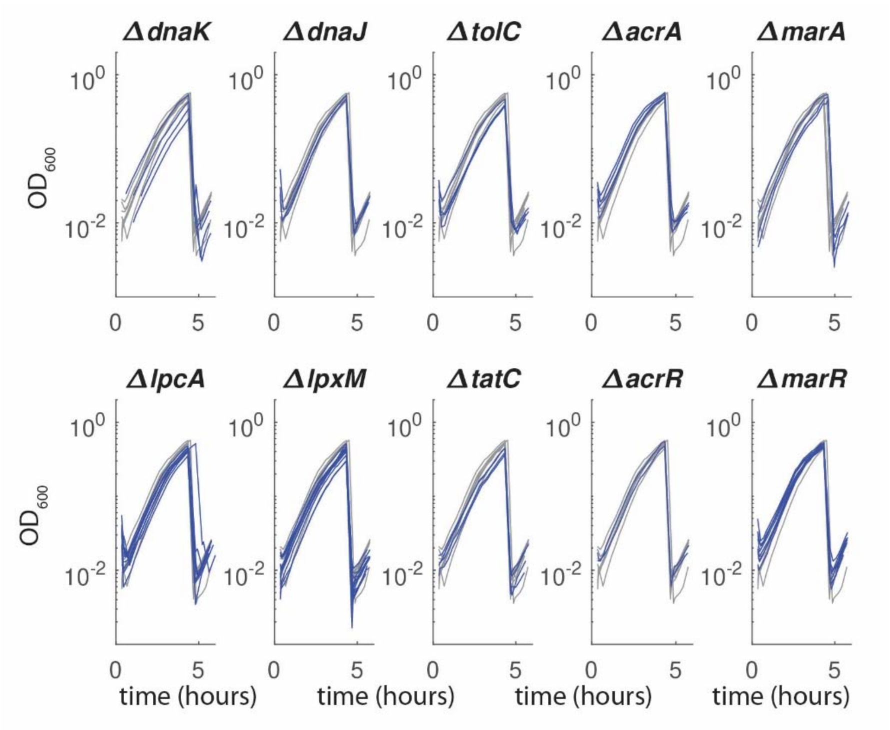

Supplementary Fig. 5 | Growth curves of selected gene deletions in antibiotic-free medium.

Each panel shows background subtracted OD_{600} values from the beginning of experiment M3. Before the dilution (at 4.5 hours), there is no antibiotic in any of the wells. After the dilution, there is a low concentration (0.15 µg ml^{-1}) of tetracycline in all wells. Gray lines show 8 replicates of the Δ*lacA* strain. All gene deletion strains grow exponentially and none of them show substantial growth defects.

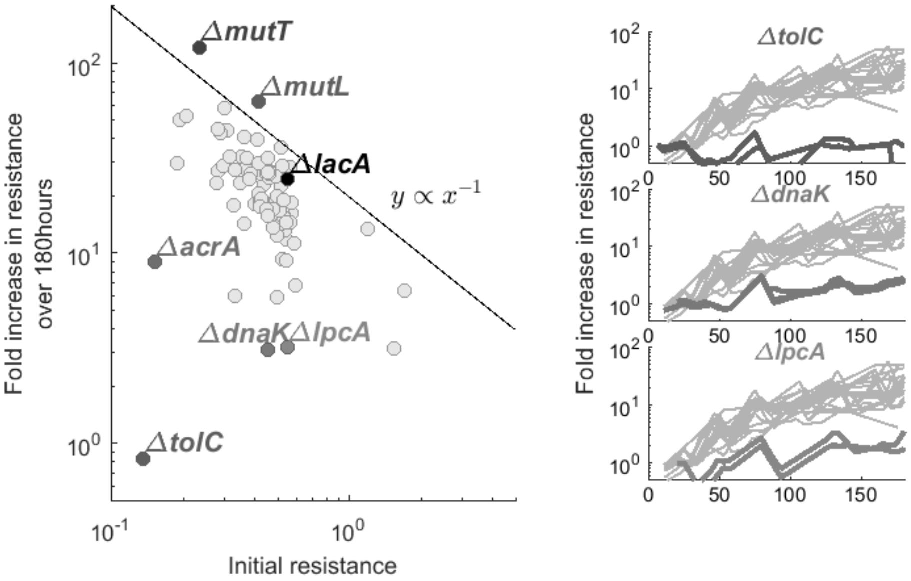

Supplementary Fig. 6 | Outliers from the diminishing returns trend.

Same plot as in Figure 2b of the main text, but with identities of notable outliers shown.

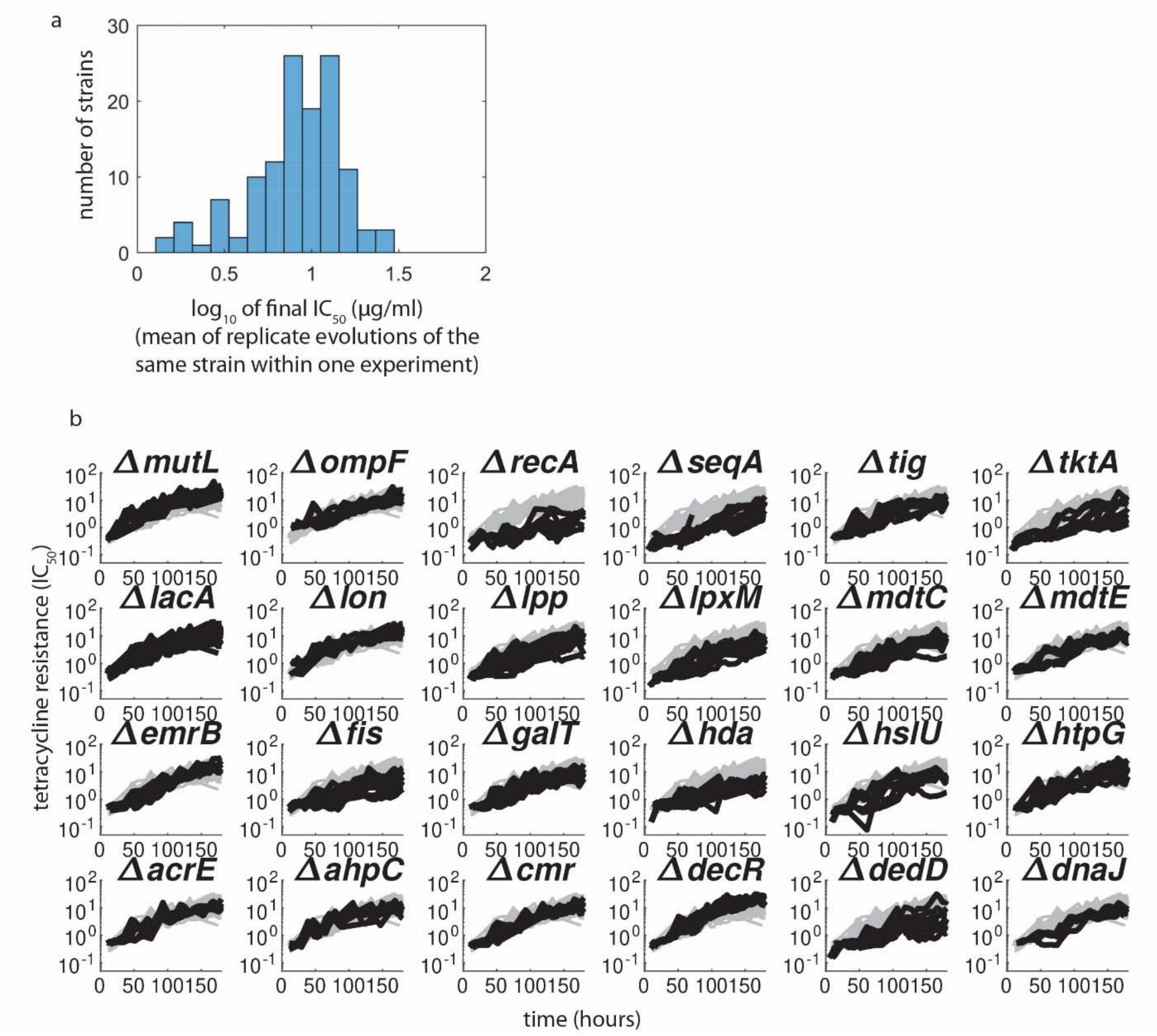

Supplementary Fig. 7 | Resistance increases over time in tetracycline for additional ancestral strains.

A) Histogram of $\log_{10}$ values of final resistance levels (tetracycline concentration in the well in µg ml^{-1}) for all strains in M2 and M3 (n=126). Each value is the mean of all evolutionary replicates for a particular strain in a particular experiment. B) Resistance (as measured by the antibiotic concentration in the well) over time for all deletion strains for which more than 3 evolutionary replicates in tetracycline were done and which were not shown in Figures 2-4. Gray lines show resistance over time for 33 replicates of the reference strain (*ΔlacA*) for comparison.

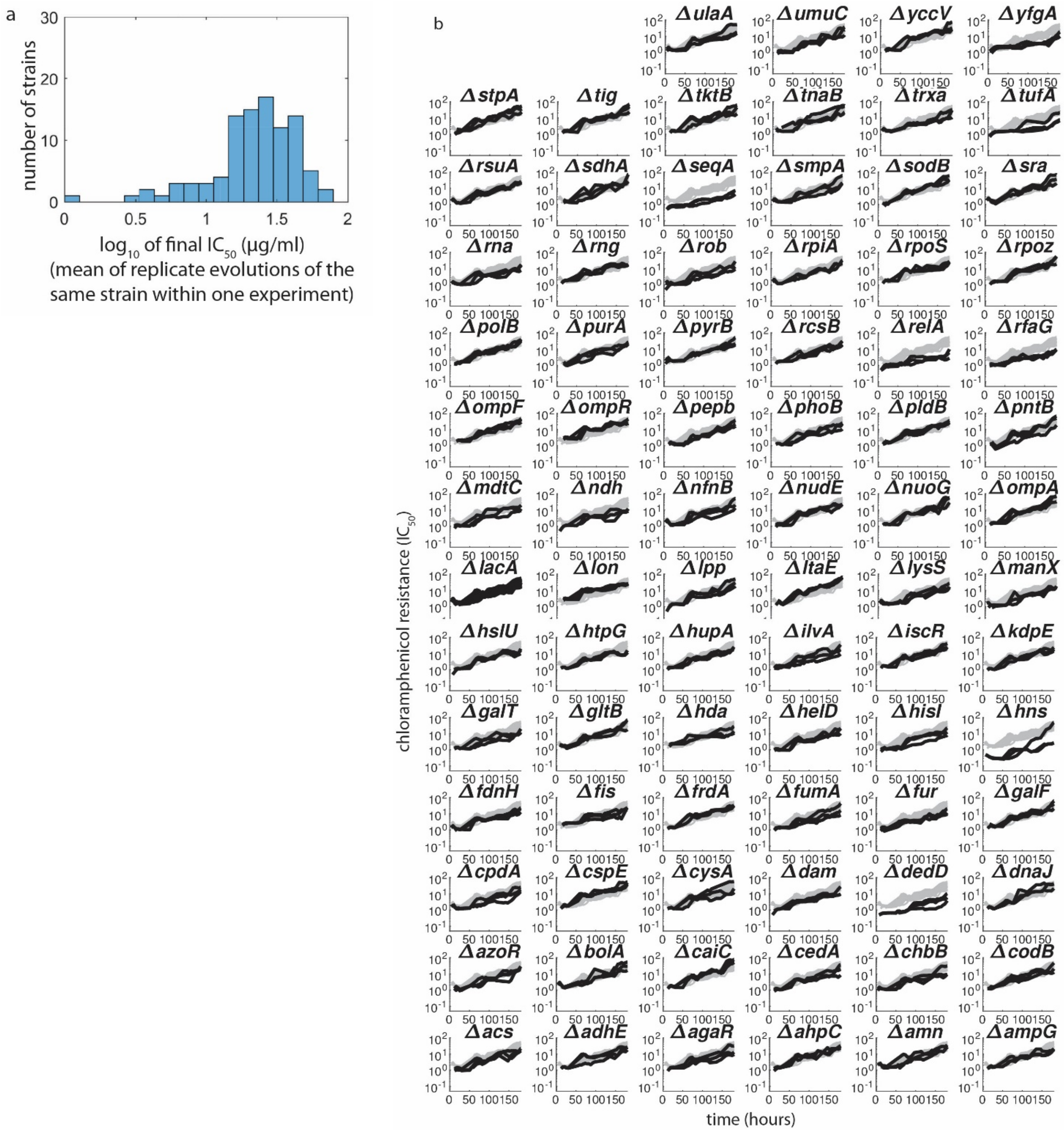

Supplementary Fig. 8 | Resistance increases over time in chloramphenicol for additional ancestral strains.

a) Histogram of $\log_{10}$ values of final resistance levels (chloramphenicol concentration in the well in µg ml^{-1}) for all strains in M4 (n=98). Each value is a mean of all evolutionary replicates for a particular strain. b) Resistance (as measured by the antibiotic concentration in the well) over time in chloramphenicol, but for all strains for which more than two replicates were done successfully (i.e. no cross-contamination was found). Gray lines show resistance over time for 23 replicates of the reference strain (*ΔlacA*) for comparison.

Experiment	Deletion strains from the Keio collection[1]
M1 – Tetracycline, short (120h) experiment	*ΔacrA, ΔacrR, ΔahpC, ΔampG, ΔatpF, ΔazoR, ΔcedA, ΔcodB, ΔcspE, ΔcysA, Δdam, ΔdedD, ΔdinB, ΔdnaK, Δfis, ΔfrdA, Δfur, ΔgalT, Δhda, ΔhisI, Δhns, ΔhslU, ΔhtpG, ΔhupA, ΔilvA, ΔkdpE, Δlon, ΔlpcA, Δlpp, ΔlpxM, ΔmarR, ΔmdtC, ΔmutL, ΔmutT, Δndh,* parent, *ΔpolB, ΔppiD, ΔproQ, ΔrecA, ΔrelA, ΔrfaG, Δrng, ΔrpoS, ΔrpsF, ΔseqA, ΔsodB, ΔtatC, ΔtolC, ΔumuC, ΔyccV*
M2 – Tetracycline	*ΔacrA, ΔacrR, Δacs, ΔadhE, ΔagaR, ΔahpC, Δamn, ΔampG, ΔastC, ΔatpF, ΔazoR, ΔbolA, ΔcaiC, ΔcedA, ΔchbB, ΔcodB, ΔcpdA, ΔcspE, ΔcysA, Δdam, ΔdedD, ΔdinB, ΔdnaK, ΔfdnH, Δfis, ΔfrdA, ΔfumA, Δfur, ΔgalF, ΔgalT, ΔgltB, Δhda, ΔhelD, ΔhisI, Δhns, ΔhslU, ΔhtpG, ΔhupA, ΔilvA, ΔiscR, ΔkdpE, ΔlacA, Δlon, ΔlpcA, Δlpp, ΔlpxM, ΔltaE, ΔlysS, ΔmanX, ΔmarR, ΔmdtC, ΔmutL, ΔmutT, Δndh, ΔnfnB, ΔnudE, ΔnuoG, ΔompA, ΔompR, Δpepb, ΔphoB, ΔpldB, ΔpntB, ΔpolB, ΔppiD, ΔproQ, ΔpurA, ΔpyrB, ΔrcsB, ΔrecA, ΔrelA, ΔrfaG, Δrna, Δrng, Δrob, ΔrpiA, ΔrplA, ΔrpoS, Δrpoz, ΔrpsF, ΔrsuA, ΔsdhA, ΔseqA, ΔsmpA, ΔsodB, Δsra, ΔstpA, ΔtatC, Δtig, ΔtktA, ΔtktB, ΔtnaB, ΔtolC, Δtrxa, ΔtufA, ΔulaA, ΔumuC, ΔyccV, ΔyfgA*
M3 – Tetracycline (fewer strains in more replicates)	*ΔacrA, ΔacrE, ΔacrR, Δcmr, ΔdedD, ΔdnaJ, ΔdnaK, ΔemrB, Δfis, ΔgalT, Δhda, ΔhslU, ΔhtpG, ΔlacA, Δlon, ΔlpcA, Δlpp, ΔlpxM, ΔmarA, ΔmarR, ΔmdtC, ΔmdtE, ΔmutL, ΔompF, ΔrecA, ΔseqA, ΔtatC, Δtig, ΔtktA, ΔtolC, ΔdecR*
M4 - Chloramphenicol	*ΔacrA, ΔacrB, ΔacrR, Δacs, ΔadhE, ΔagaR, ΔahpC, Δamn, ΔampG, ΔastC, ΔatpF, ΔazoR, ΔbolA, ΔcaiC, ΔcedA, ΔchbB, Δcmr, ΔcodB, ΔcpdA, ΔcspE, ΔcysA, Δdam, ΔdedD, ΔdinB, ΔdnaJ, ΔdnaK, ΔfdnH, Δfis, ΔfrdA, ΔfumA, Δfur, ΔgalF, ΔgalT, ΔgltB, Δhda, ΔhelD, ΔhisI, Δhns, ΔhslU, ΔhtpG, ΔhupA, ΔilvA, ΔiscR, ΔkdpE, ΔlacA, Δlon, ΔlpcA, Δlpp, ΔlpxM, ΔltaE, ΔlysS, ΔmanX, ΔmarA, ΔmarR, ΔmdtC, ΔmutL, ΔmutT, Δndh, ΔnfnB, ΔnudE, ΔnuoG, ΔompA, ΔompF, ΔompR, Δpepb, ΔphoB, ΔpldB, ΔpntB, ΔpolB, ΔppiD, ΔproQ, ΔpurA, ΔpyrB, ΔrcsB, ΔrecA, ΔrelA, ΔrfaG, Δrna, Δrng, Δrob, ΔrpiA, ΔrplA, ΔrpoS, Δrpoz, ΔrpsF, ΔrsuA, ΔsdhA, ΔseqA, ΔsmpA, ΔsodB, Δsra, ΔstpA, ΔtatC, Δtig, ΔtktA, ΔtktB, ΔtnaB, ΔtolC, Δtrxa, ΔtufA, ΔulaA, ΔumuC, ΔyccV, ΔyfgA*

Supplementary Table 1 | Lists of strains used in evolution experiments.

	M1	M2	M3	M4
Antibiotic	Tetracycline	Tetracycline	Tetracycline	Chloramphenicol
Number of different strains	51	99	31	104
Replicates per strain	3	3	8-12	3
Control strain	BW25113 (Keio parent)	Δ*lacA*	Δ*lacA*	Δ*lacA*
Replicates of control	10	22	11	23
Medium	LB	LB+50μg ml^{-1} kanamycin	LB+50μg ml^{-1} kanamycin	LB+50μg ml^{-1} kanamycin

Supplementary Table 2 | Differences between instances of the automated evolution experiments.

	Estimate	Standard error	t-statistic	p-value
b0	1.4104	0.18995	7.4253	$1.03 \cdot 10^{-11}$
b1 (marR)	1.171	0.17571	6.6641	$5.75 \cdot 10^{-10}$
b2 (*lon*)	1.1078	0.17225	6.4311	$1.90 \cdot 10^{-9}$
b3 (pumps)	0.3859	0.19225	2.0073	0.046656
b4 (*acrR*)	0.43499	0.19603	2.219	0.028108
b5 (*decR*)	0.68787	0.18469	3.7245	0.00028361

Supplementary Table 3 | Estimates of the resistance contributions of the five most common mutations based on a linear regression model (number of observations: 145, Error degrees of freedom: 139, Adjusted R^2 : 0.533, F-statistic vs. constant model: 33.8, p-value = $1.73 \cdot 10^{-22}$, Methods). One-sided t-tests were performed for each coefficient to test the null hypothesis that the coefficient is zero given the other coefficients in the model; the p-values from these tests are shown in the right-most column.

Oligo ID	locus	Forward (5‘ to 3‘)	Reverse (5‘-3‘)
Primer pairs for genotype verification after P1 transduction			
23,24	*lacA*	GGTTCCTTACTGGCATTG	GCTGGAAGTGGTTATTCTG
25,26	*tolC*	CGCGCTAAATACTGCTTC	GTTGCCTTACGTTCAGACG
27,28	*dnaK*	CACAACCACATGATGACC	CCTAGATGAATGCACGGG
Primer pair for construction of Δ*lpcA*::*kanR*			
21,22	*lpcA*	CGGTACACTGCATTTTGTCTATTACATTTATGCTGAAGGATATCCTCATGattccggggatccgtcgacc	GTAAACGTCTTATCCGGCCTACGCCAGACTTACTTAACCATCTCTTTTTCtgtaggctggagctgcttcg
Primer pair for colony PCR			
35,36	*lpcA*	CGGTACACTGCATTTTGTCT	GGCGTAAACGTCTTATCCGG

Supplementary Table 4 | Primer pairs used for construction and verification of double deletion strains.

Supplementary Reference:

1. Baba, T. *et al.* Construction of Escherichia coli K-12 in-frame, single-gene knockout mutants: the Keio collection. *Mol. Syst. Biol.* **2**, 2006.0008 (2006).
