# Supplementary material for: Highly parallel lab evolution reveals that epistasis can curb the evolution of antibiotic resistance
Source: Nat Commun. 2020 Jun 19;11:3105. doi: 10.1038/s41467-020-16932-z (PMC7305214; doi:10.1038/s41467-020-16932-z)
Supplement: Supplementary file 3 — Description of Additional Supplementary Files [file 41467_2020_16932_MOESM3_ESM.pdf]

Description of Additional Supplementary Files

File Name: Supplementary Data 1

Description: **Descriptions of all fixed mutations found in ancestral and evolved clones.** For amplifications, the approximate amplification factor is shown in column ‘amplification’. Abbreviations: del – deletion; IS – IS element; ins – insertion; snp – single nucleotide polymorphism; amp – amplification.
