# Supplementary material for: Highly parallel lab evolution reveals that epistasis can curb the evolution of antibiotic resistance
Source: Nat Commun. 2020 Jun 19;11:3105. doi: 10.1038/s41467-020-16932-z (PMC7305214; doi:10.1038/s41467-020-16932-z)
Supplement: Supplementary file 5 — Reporting Summary [file 41467_2020_16932_MOESM5_ESM.pdf]

nature research

Corresponding author(s): Tobias Bollenbach

Last updated by author(s): May 20, 2020

Reporting Summary

Nature Research wishes to improve the reproducibility of the work that we publish. This form provides structure for consistency and transparency in reporting. For further information on Nature Research policies, see Authors & Referees and the Editorial Policy Checklist .

Statistics

For all statistical analyses, confirm that the following items are present in the figure legend, table legend, main text, or Methods section.

n/a	Confirmed	
☐	☒	The exact sample size (*n*) for each experimental group/condition, given as a discrete number and unit of measurement
☐	☒	A statement on whether measurements were taken from distinct samples or whether the same sample was measured repeatedly
☐	☒	The statistical test(s) used AND whether they are one- or two-sided<br>*Only common tests should be described solely by name; describe more complex techniques in the Methods section.*
☐	☒	A description of all covariates tested
☐	☒	A description of any assumptions or corrections, such as tests of normality and adjustment for multiple comparisons
☐	☒	A full description of the statistical parameters including central tendency (e.g. means) or other basic estimates (e.g. regression coefficient) AND variation (e.g. standard deviation) or associated estimates of uncertainty (e.g. confidence intervals)
☐	☒	For null hypothesis testing, the test statistic (e.g. *F*, *t*, *r*) with confidence intervals, effect sizes, degrees of freedom and *P* value noted<br>*Give P values as exact values whenever suitable.*
☒	☐	For Bayesian analysis, information on the choice of priors and Markov chain Monte Carlo settings
☒	☐	For hierarchical and complex designs, identification of the appropriate level for tests and full reporting of outcomes
☐	☒	Estimates of effect sizes (e.g. Cohen's *d*, Pearson's *r*), indicating how they were calculated

Our web collection on statistics for biologists contains articles on many of the points above.

Software and code

Policy information about availability of computer code

Data collection	Customized Freedom EVOware® scripts were used to implement automatized evolution experiments. A Python 2.6.9. script was used to calculate growth rates and control the dynamic feedback in the protocol. Details of these scripts are included in the Methods section of the manuscript.
Data analysis	Simple Matlab R2016b scripts were used to organize, visualize and analyze the experimental data. All relevant functions are described in the Methods section of the manuscript. Breseq software (version 0.32.0) was used to call mutations from sequencing data. Breseq utilizes bowtie (version 1.2.1.1) to align the short reads to the reference.

For manuscripts utilizing custom algorithms or software that are central to the research but not yet described in published literature, software must be made available to editors/reviewers. We strongly encourage code deposition in a community repository (e.g. GitHub). See the Nature Research guidelines for submitting code & software for further information.

Data

Policy information about availability of data

All manuscripts must include a data availability statement. This statement should provide the following information, where applicable:
- Accession codes, unique identifiers, or web links for publicly available datasets
- A list of figures that have associated raw data
- A description of any restrictions on data availability

Whole-genome sequencing data is accessible in the European Nucleotide Archive under accession code PRJEB37495 [https://www.ebi.ac.uk/ena/data/view/PRJEB37495]. All other data is included within the main text or supplementary materials. Figures 3b, 6a, Supplementary Figures 2 and 3 are built on the sequencing data and resulting mutation calls available in Supplementary Data 1. Data to produce all other figures is available in Supplementary Data 2.

Field-specific reporting

Please select the one below that is the best fit for your research. If you are not sure, read the appropriate sections before making your selection.

- [x] Life sciences
- [ ] Behavioural & social sciences
- [ ] Ecological, evolutionary & environmental sciences

For a reference copy of the document with all sections, see nature.com/documents/nr-reporting-summary-flat.pdf

Life sciences study design

All studies must disclose on these points even when the disclosure is negative.

Sample size	The number of different ancestral (gene deletion) strains used in evolution experiments was chosen to allow for representatives of major cellular pathways that are expressed in our conditions and meet the limit of parallel experiments we could perform on our platform. Evolution experiments were performed in at least three replicates; this was sufficient since the resistance phenotypes of replicates were generally consistent across replicates as can be seen in Fig1e. Selected evolution experiments were performed in up to 23 replicates to assess the repeatability of resistance mutations.
Data exclusions	Samples were excluded from analysis if contamination of the sample was detected by targeted PCR or whole-genome sequencing. The full list of experiments that were excluded due to these reasons is given in Supplementary Data 2 (Sheet: 'ExcludedExperiments').
Replication	Parallel experiments were conducted at least in triplicate; only small differences in results between replicates and instances of experiments repeated at a later time were observed. The number of replicate experiments performed for each condition is given in Supplementary Table 2.
Randomization	There was no classical allocation into distinct experimental groups in our study.
Blinding	There was no classical allocation into distinct experimental groups in our study: hundreds of experiments were performed in parallel by a computer-controlled robotic system that has no information about the specific experiment that is running and treats all experiments identically. The same applies to the data analysis: the entire analysis was automated and is applied identically to all experiments.

Reporting for specific materials, systems and methods

We require information from authors about some types of materials, experimental systems and methods used in many studies. Here, indicate whether each material, system or method listed is relevant to your study. If you are not sure if a list item applies to your research, read the appropriate section before selecting a response.

Materials & experimental systems

n/a	Involved in the study
x	Antibodies
x	Eukaryotic cell lines
x	Palaeontology
x	Animals and other organisms
x	Human research participants
x	Clinical data

Methods

n/a	Involved in the study
x	ChIP-seq
x	Flow cytometry
x	MRI-based neuroimaging
