# Supplementary material for: Highly parallel lab evolution reveals that epistasis can curb the evolution of antibiotic resistance
Source: Nat Commun. 2020 Jun 19;11:3105. doi: 10.1038/s41467-020-16932-z (PMC7305214; doi:10.1038/s41467-020-16932-z)
Supplement: Supplementary file 2 — Peer Review File [file 41467_2020_16932_MOESM2_ESM.pdf]

Editorial Note: This manuscript has been previously reviewed at another journal that is not operating a transparent peer review scheme. The manuscript was considered suitable for publication without further review at *Nature Communications*.
